# RNA sequencing reveals changes in the microRNAome of transdifferentiating hepatic stellate cells that are conserved between human and rat

**DOI:** 10.1038/s41598-020-78776-3

**Published:** 2020-12-10

**Authors:** Laura Sabater, Luigi Locatelli, Fiona Oakley, Timothy Hardy, Jeremy French, Stuart M. Robinson, Gourab Sen, D. A. Mann, Jelena Mann

**Affiliations:** 1 Newcastle Fibrosis Research Group, Bioscience Institute, Faculty of Medical Sciences, Newcastle University, 4th Floor, William Leech Building, Framlington Place, Newcastle upon Tyne, NE2 4HH UK; 2 Department of Hepatobiliary Surgery, Newcastle Upon Tyne Hospitals NHS Foundation Trust, Newcastle upon Tyne, UK

**Keywords:** Epigenetics, Non-coding RNAs

## Abstract

MicroRNAs are small (~ 22nt long) noncoding RNAs (ncRNAs) that regulate gene expression at the post-transcriptional level. Over 2000 microRNAs have been described in humans and many are implicated in human pathologies including tissue fibrosis. Hepatic stellate cells (HSC) are the major cellular contributors to excess extracellular matrix deposition in the diseased liver and as such are important in the progression of liver fibrosis. We employed next generation sequencing to map alterations in the expression of microRNAs occurring across a detailed time course of culture-induced transdifferentiation of primary human HSC, this a key event in fibrogenesis. Furthermore, we compared profiling of human HSC microRNAs with that of rat HSC so as to identify those molecules that are conserved with respect to modulation of expression. Our analysis reveals that a total of 229 human microRNAs display altered expression as a consequence of HSC transdifferentiation and of these 104 were modulated early during the initiation phase. Typically modulated microRNAs were targeting kinases, transcription factors, chromatin factors, cell cycle regulators and growth factors. 162 microRNAs changed in expression during transdifferentiation of rat HSC, however only 17 underwent changes that were conserved in human HSC. Our study therefore identifies widespread changes in the expression of HSC microRNAs in fibrogenesis, but suggests a need for caution when translating data obtained from rodent HSC to events occurring in human cells.

## Introduction

Fibrotic diseases underlie about 45% of all chronic diseases and deaths in the developed world^1^. Hepatic fibrosis is a progressive pathology characterised by a dysregulated wound-healing response caused by chronic liver injury of various aetiologies, including alcoholic liver disease (ALD), chronic viral infection and non-alcoholic fatty liver disease (NAFLD) among others. This uncontrolled wound-healing response is a highly dynamic process characterized by the accumulation of extracellular matrix (ECM) which reflects the imbalance between liver repair and scar formation^2^. Fibrogenesis is chiefly driven by hepatic stellate cells (HSC) which act as a local source of fibrogenic myofibroblasts. In the absence of liver damage, HSC reside in the space of Dissé in a quiescent state where their main function is to store. However, after a liver insult occurs, HSC undergo transdifferentiation into highly proliferative, collagen-secreting and contractile myofibroblast-like phenotype often referred to as the “activated HSC”^3^. The process of HSC transdifferentiation was described more than 15 years ago to have two distinct phases^4^. The early phase, which involves epigenetic and transcriptional re-landscaping of the HSC is known as ‘initiation’ and is in response to paracrine signals emanating from damaged hepatocytes and activated Kupffer cells. At the end of “initiation” the HSC is transformed into a cell that can produce collagen and proliferate in response to autocrine as well as paracrine stimuli, this transitions to the second phase of “perpetuation’ which involves amplification of the activated phenotype with increased cytokine expression, upregulation of key tyrosine kinase receptors and an acceleration of ECM remodelling^2,3^. Furthermore, morphological differences have been observed in HSC from these two different stages of transdifferentiation^5^.

MicroRNAs (miRNAs) are small noncoding RNAs (~ 22nt long) that regulate gene expression at the post-transcriptional level, thus playing an important role in the control of a plethora of developmental, physiological and pathophysiological processes^6^. Cumulative evidence has shown that miRNAs are involved in many different aspects of liver fibrosis, including HSC activation. Expression analyses have highlighted miRNA-21^7^, miRNA-221 and miRNA-222^8^ as being upregulated during HSC transdifferentiation, whereas miRNA-122^9^, miRNA-150^10^, miRNA-194^10^ and miRNA-132^11^ undergo downregulation of their expression in the activated HSC. Although these miRNAs and others have been described as putative regulators of the HSC phenotype, we are lacking a systematic analysis of the dynamic changes in miRNA expression during the two phases of transdifferentiation. Furthermore, there is a paucity of knowledge regarding the degree to which remodelling of the miRNA landscape compares between rodent and human HSC, this information being important for identifying miRNAs that are likely to play fundamental conserved roles in fibrogenesis.

In this study we perform a global miRNA analysis across a detailed time-course of culture-induced transdifferentiation of isolated rat and human HSC. Our results show substantial remodelling of the miRNA expression landscape between HSC “initiation” and “perpetuation”, which suggests that alterations in the expression of particular miRNAs may facilitate phenotypic transitions towards the myofibroblast state. Moreover, some of the observed changes were consistent between rat and human HSC. Our findings provide new insights into the complexities of the process that govern HSC activation and the importance for the discovery of new markers for the early stages of HSC activation.

## Results

**Figure 1:**
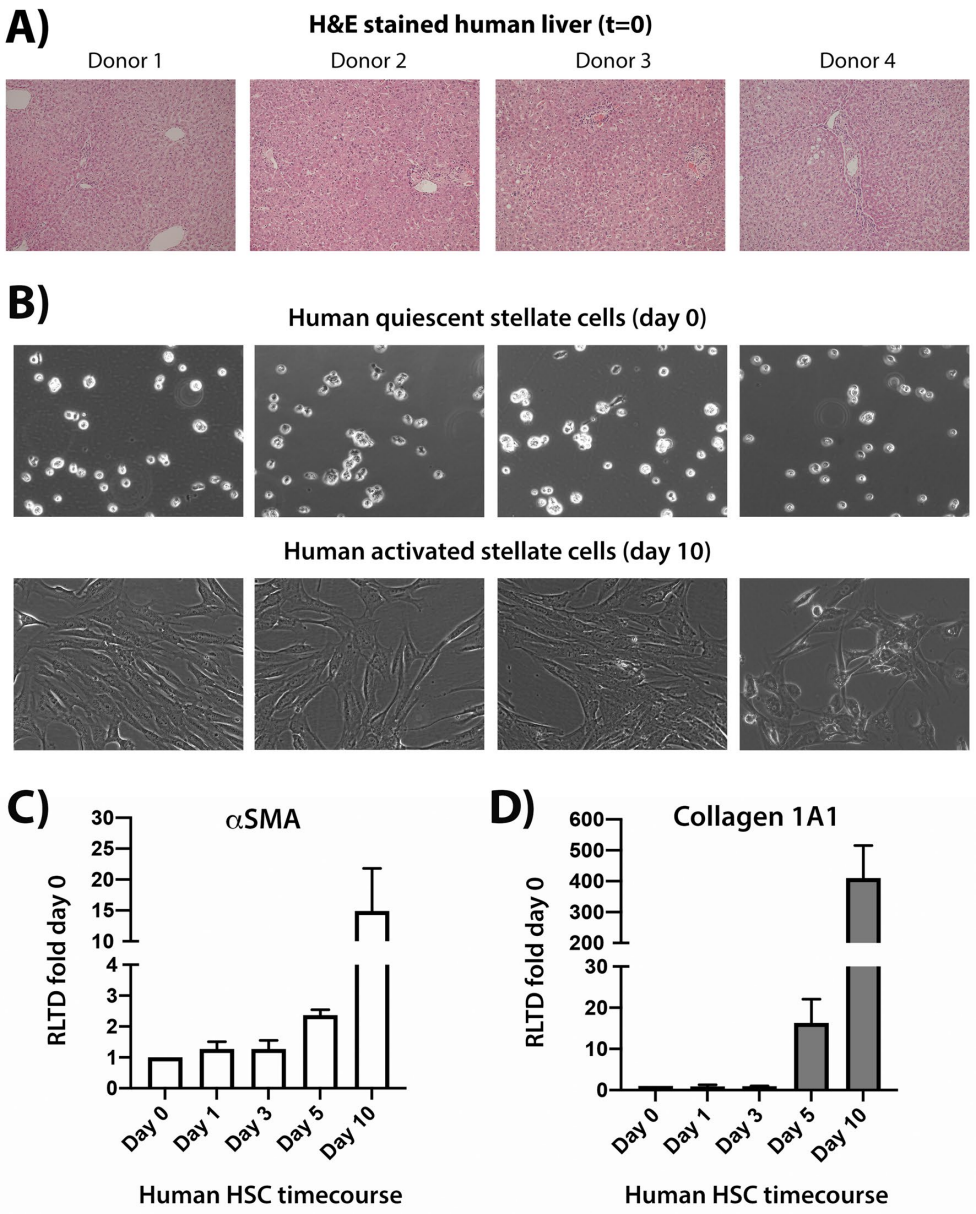
Human HSC isolated from normal human livers and timecourse of their in vitro activation. (**A**) Representative brightfield H&E stained images of four human donor livers used in the study, which depict normal tissue structure and architecture at X10 magnification. (**B**) Representative brightfield images of four human HSC preps used in the study showing quiescent HSC in top panel and fully activated HSC in the bottom panel. (**C**) Quantitative PCR carried out on time-course cDNA isolated from of human HSC preps used in the study showing αSMA transcript levels in (**C**) and Collagen1A1 in (**D**).

To systematically determine global changes in miRNA expression during culture-induced transdifferentiation of human HSC, we isolated HSC from four human donor livers (Fig. 1A), which were either immediately harvested (day 0 or freshly isolated, Fig. 1B, top panel), or were cultured on plastic in serum-containing media for 1, 3, 5 and 10 days (Fig. 1B, bottom panel) prior to harvesting to generate a time-course of HSC transdifferentiation. Timecourse induced activation status of HSC was confirmed by measuring alpha smooth muscle actin (αSMA) (Fig. 1C), Collagen1A1 gene expression levels (Fig. 1D) as well as TGFβ1 and TIMP-1 (Supplementary Fig. 1).

### Changes in miRs during early activation of human HSC

**Figure 2:**
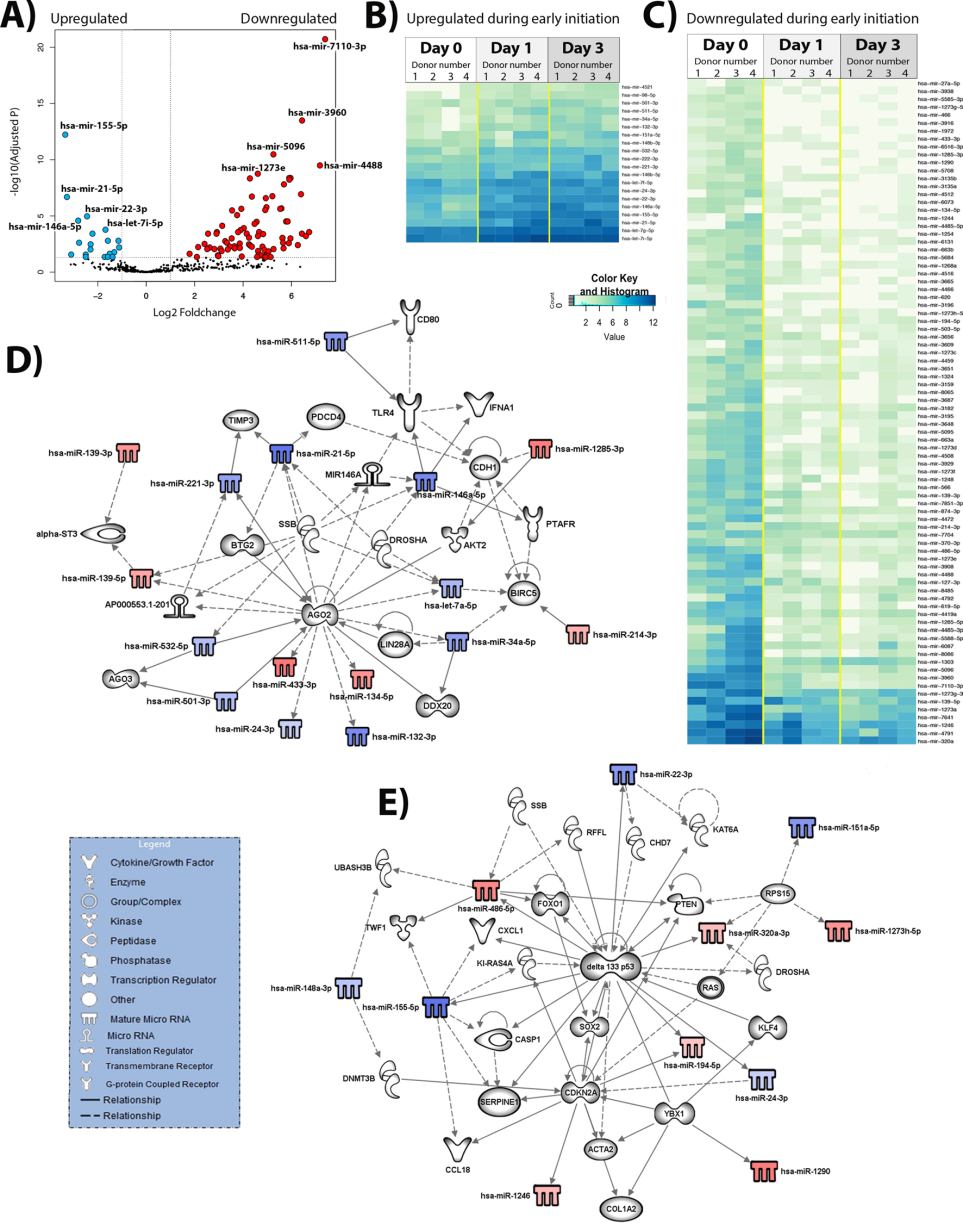
Differential expression of human HSC activation during the initiation stage. (**A**) Volcano plot of the comparison between the freshly isolated, quiescent HSC (day 0, n = 4) and day 3 HSC (initiation of activation, day 3, n = 4). In blue—differentially under-expressed miRNA in day 0; in red—differentially over-expressed miRNA in day 0 (p-adjusted < 0.05 and foldchange > 1). (**B**) Heatmap of the 20 significantly under-expressed miRNA in day 0 when compared to day 3, p-adjusted < 0.05. (**C**) Heatmap of the 84 significantly over-expressed miRNA in day 0 when compared to day 3, p-adjusted < 0.05. (**D**) and (**E**) Biological networks of differentially expressed miRNA. In blue and red, under and over-expressed miRNA, respectively. Shadowed in grey are molecules related to miRNA, as displayed on the legend. Lines represent different relationships between the molecules and miRNA.

Following cell harvesting, miRNAs were purified from which next generation sequencing (NGS) libraries prepared and subsequently sequenced using the MiSeq Illumina platform. Days 0, 1 and 3 were considered to represent the early “initiation” phase of transdifferentiation, with time-points day 5 and 10 representing the perpetuation to fully activated states. Figure 2A shows a volcano plot and associated heatmaps (Fig. 2B,C) displaying 20 upregulated and 84 downregulated miRNA species, relative to day 0, identified from the NGS as reporting a significant change in expression between day 0, 1 and 3 cultured HSC (Supplementary Table 1). Ingenuity pathway analysis (IPA) of the differentially expressed miRNAs indicates putative functions in the early induction of fibrogenesis as evidenced by effects on a number of regulatory genes including kinases (TWF1, Akt2), translation regulators involved in microRNA and siRNA processing (Ago2, Ago3, Drosha), transcriptional regulators (KLF4, YBX1, Sox2, FOXO1, delta133p53, DDX20, BTG2), chromatin regulating factors (DNMT3B, KAT6A), cytokine/growth factor complexes (CXCL1, CCL18, IFNA1) (Fig. 2D). In addition, modulated miRNAs were identified that mapped to well documented profibrogenic genes including Acta2 which codes for alpha smooth muscle actin (αSMA) and Collagen 1A2 (Fig. 2E). Hence changes in the expression of multiple miRNAs occurs very early in HSC transdifferentiation and may impact on several important regulatory pathways relevant to fibrogenesis.

### Changes in miR s during perpetuation/full activation of human HSC

**Figure 3:**
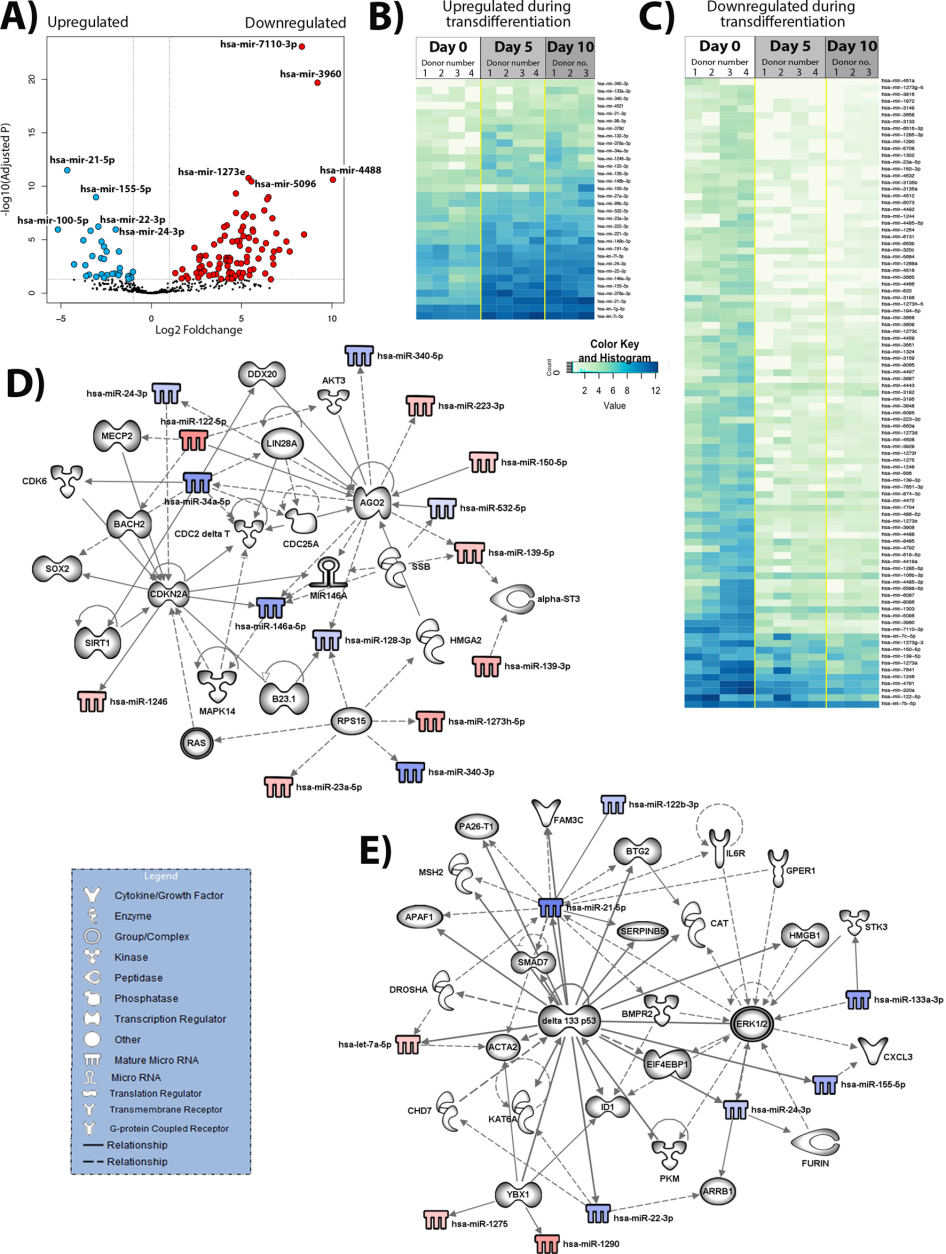
Differential expression of human HSC activation during the perpetuation stage. (**A**) Volcano plot of the comparison between the freshly isolated, quiescent HSC (day 0, n = 4) and fully activated HSC (day 10, n = 3). In blue—differentially under-expressed miRNA in day 0; in red—differently over-expressed miRNA in day 0 (p-adjusted < 0.05 and foldchange > 1). (**B**) Heatmap of the 32 significant under-expressed miRNA in day 0 when compared to day 10, p-adjusted < 0.05. (**C**) Heatmap of the 93 significant miRNA over-expressed in day 0 when compared to day 10, p-adjusted < 0.05. (**D**) and (**E**) Biological networks of differentially expressed miRNA. In blue and red, under and over-expressed miRNA, respectively. Shadowed in grey are molecules related to miRNA, as displayed on the legend. Lines represent different relationships between the molecules and miRNA.

We next compared the miRNA profile of freshly isolated (day 0) HSC with that found at culture days 5 and 10 which represent the “activated” myofibroblast HSC phenotype. 32 upregulated and 93 downregulated miRNA species, relative to day 0, were identified (Fig. 3A–C, Supplementary Table 2) that mapped to pathways involving similar gene categories as outlined for early initiation, but included a number of additional genes. We identified alterations in miRNAs with the potential to influence kinases (CDK6, CDC2deltaT, MAPK14, BMPR2, PKM, STK3, Akt3), a translation regulator (EIF4EBP1), multiple key epigenetic and transcriptional regulators (MeCP2, BACH2, SIRT1, CDKN2A, HMGB1, B23.1, ID1), enzymes/chromatin regulators (CAT (catalase), MSH2 (DNA repair), HMGA2, CHD7 (DNA helicase)), cytokine/growth factor complexes (CXCL3, FAM3C) and complexes involved in Ras and ERK1/2 pathways (Fig. 3D,E). Taken together, these data suggest there are overlapping as well as distinct mechanisms operating in different phases of HSC transdifferentiation, which appear to be affected by associated changes in the miRNA landscape.

### Changes in miRs during activation of rat HSC

**Figure 4:**
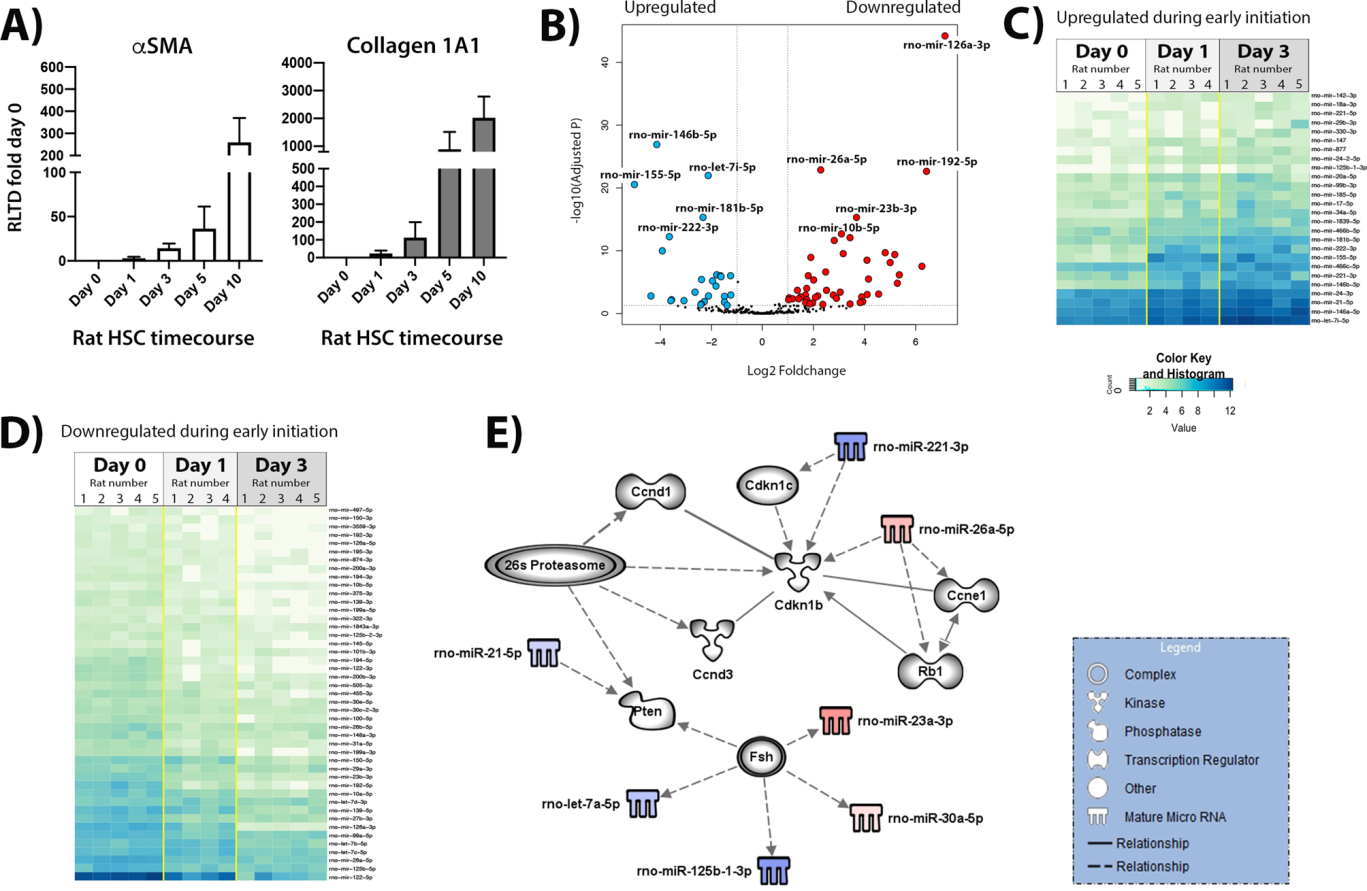
Differential expression of rat HSC activation during the initiation stage. (**A**) Quantitative PCR carried out on time-course cDNA isolated from of rat HSC preps used in the study showing αSMA transcript levels in left panel and Collagen1A1 in right panel. (**B**) Volcano plot of the comparison between the quiescent HSC (day 0, n = 5) and day 3 HSC (initiation of activation, day 3, n = 5). In blue—differentially under-expressed miRNA in day 0; in red—differentially over-expressed miRNA in day 0 (p-adjusted < 0.05 and foldchange > 1). (**C**) Heatmap of the 26 significant under-expressed miRNA in day 0 when compared to day 3, p-adjusted < 0.05. (**D**) Heatmap of the 45 significant miRNA over-expressed in day 0 when compared to day 3, p-adjusted < 0.05. (**E**) Biological networks of the differentially expressed miRNA. In blue and red, under and over-expressed miRNA, respectively. Shadowed in grey are molecules related to miRNA, as displayed on the legend. Lines represent different relationships between the molecules and miRNA.

**Figure 5:**
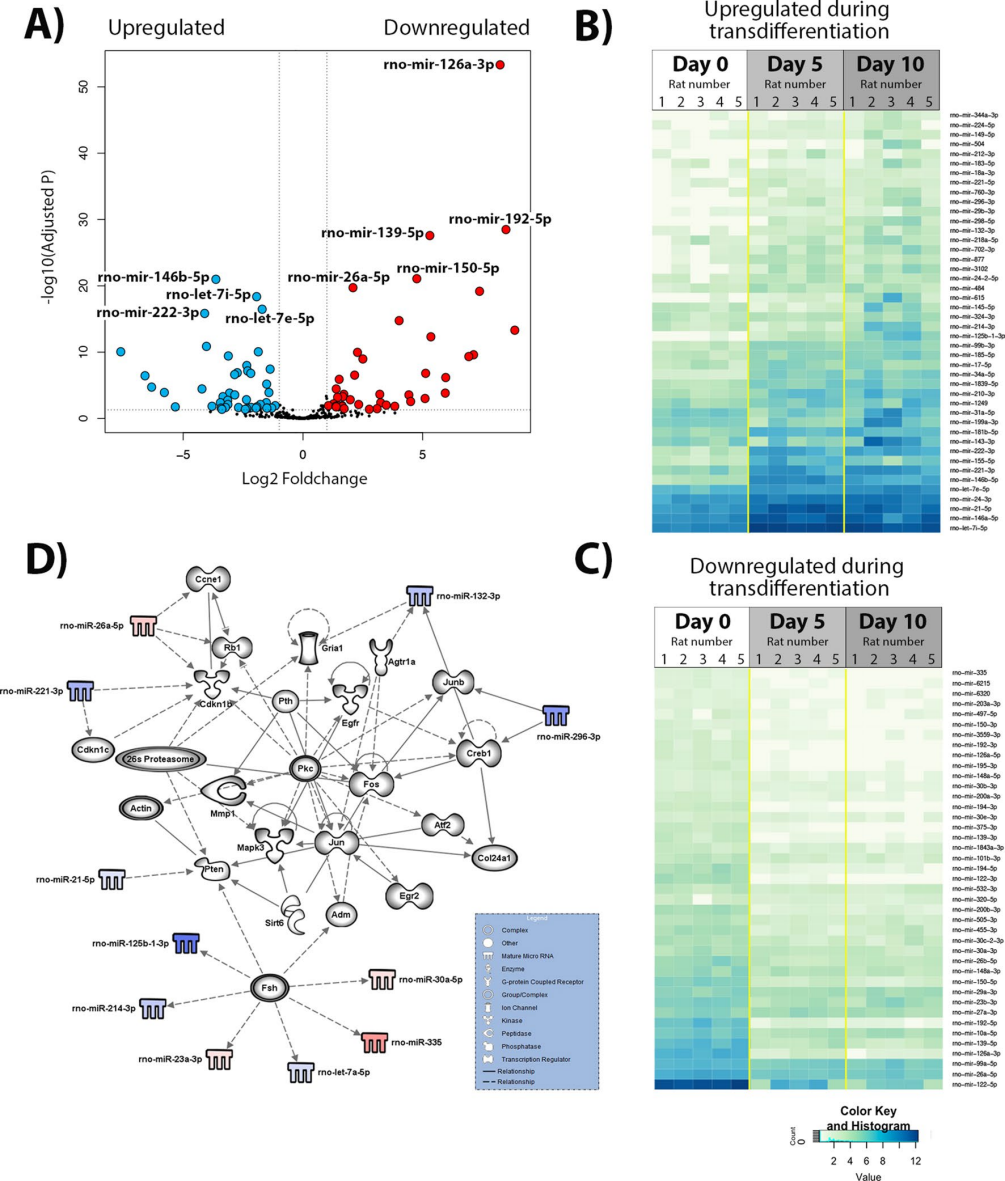
Differential expression of rat HSC activation during the perpetuation stage. (**A**) Volcano plot of the comparison between the quiescent HSC (day 0, n = 5) and fully activated HSC (day 10, n = 5). In blue—differentially under-expressed miRNA in day 0; in red—differentially over-expressed miRNA in day 0 (p-adjusted < 0.05 and foldchange > 1). (**B**) Heatmap of the 44 significant under-expressed miRNA in day 0 when compared to day 10, p-adjusted < 0.05. (**C**) Heatmap of the 41 significant miRNA over-expressed in day 0 when compared to day 10, p-adjusted < 0.05. (**D**) Biological network of differentially expressed miRNA. In blue and red, under and over-expressed miRNA, respectively. Shadowed in grey are molecules related to miRNA, as displayed on the legend. Lines represent different relationships between the molecules and miRNA.

We next focused on how the miRNA landscape changes in rat HSC over a similar time course of culture-induced transdifferentiation. Activation of rat HSC was confirmed by measuring αSMA (Fig. 4A, left panel) and Collagen1A1 gene expression levels (Fig. 4A, right panel). The purpose of this investigation was to delineate common as well as species-dependent alterations in miRNA expression and which may uncover genes and pathways that are crucial to the HSC activation process irrespective of the species. A volcano plot of miRNAs detected in NGS sequencing and subsequent mapping of differentially regulated transcripts revealed that the early phase of transdifferentiation of rat HSC (day 0 to days 1 and 3) is accompanied by altered expression of 71 miRNAs of which 26 species were upregulated and 45 downregulated relative to day 0 (Fig. 4B–D and Supplementary Table 3). Differentially expressed miRNAs appear to be involved in controlling cell cycle and proliferation, which is highly upregulated as a key defining functional feature of the activated HSC. Figure 4E shows miRNAs that may influence multiple cell cycle regulators including cyclin D1 (Ccnd1), cyclin D3 (Ccnd3), cyclin E1 (Ccne1) and Rb1 (RB Transcriptional Corepressor 1). When we compared the miRNA profile of quiescent rat HSC with that found in day 5 and 10 activated myofibroblasts, there were 44 upregulated and 41 downregulated miRNA species relative to day 0 (Fig. 5A–C, Supplementary Table 4). These miRNAs have putative targets that regulate pathways involving gene transcription (Jun, Fos, JunB, Creb1, Atf2, Rb1, Ccne1, Egr2), activities of protein kinases (Egfr, Cdkn1b, Mapk3, PKC), acetyltransferases (Sirt6) and control of the 26S proteasome complex among others (Fig. 5D). Modulation of RNA regulators of these pathways is in keeping with the need for the fully activated HSC to respond to a wide variety of extracellular paracrine and autocrine stimuli.

### Differentially expressed miRs correlated in both species

**Figure 6:**
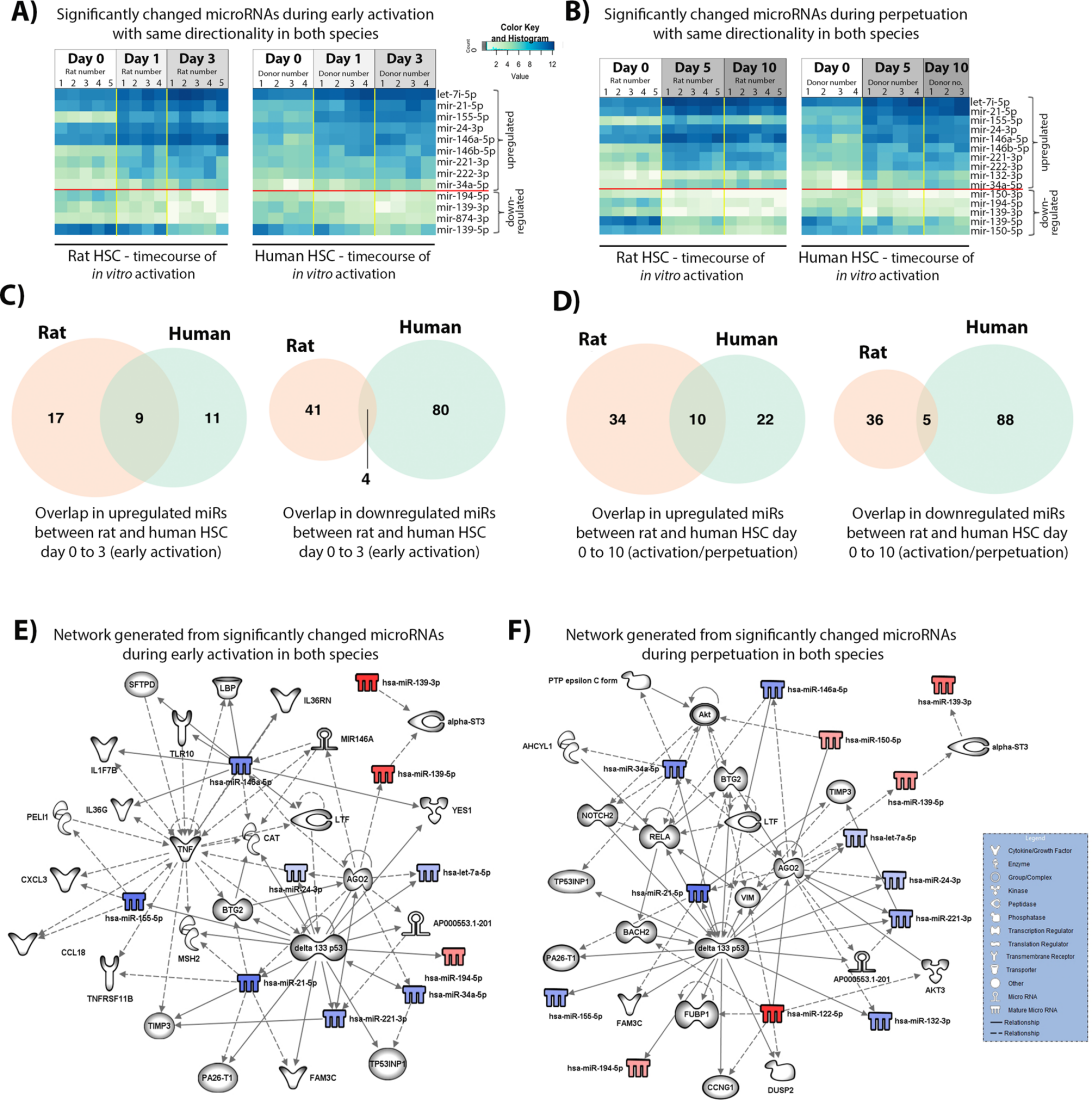
Significantly different miRNA with the same directionality of expression change in both rat and human species. (**A**) Heatmaps of the significantly changed miRNA during the initiation stage of HSC activation in rat (left) and human (right). (**B**) Heatmaps of the significantly changed miRNA during the perpetuation stage in rat (left) and human (right). (**C**) Venn diagram showing the number of significantly upregulated (left) or downregulated (right) miRs during the initiation stage of HSC activation in rat and human. (**D**) Venn diagram showing the number of significantly upregulated (left) or downregulated (right) miRs during the perpetuation/full activation stage of HSC activation in rat and human. (**E**) Biological network of significantly differentially expressed miRNA with the same directionality in both species during the early stages of HSC activation. In blue and red, under and over-expressed miRNA, respectively. Shadowed in grey are molecules related to miRNA, as displayed on the legend. Lines represent different relationships between the molecules and miRNA. (**F**) Biological network of significantly differentially expressed miRNA with the same directionality in both species during the perpetuation of HSC activation. In blue and red, under and over-expressed miRNA, respectively. Shadowed in grey are molecules related to miRNA, as displayed on the legend. Lines represent different relationships between the molecules and miRNA. Networks were generated using miRNA from the overlapping between rat and human, however I am not sure how to explain in a fig legend).

The data we obtained allowed us to carry out a number of additional comparisons in order to ascertain the miRNAs that change with same temporality and directionality (i.e. either up or downregulated) in both species. We reasoned that this may uncover miRNAs and affected pathways that are essential to the process of HSC transdifferentiation in a species independent manner. These analyses have led us to uncover 13 miRNAs that change in same direction in both species during early HSC activation (upregulated—hsa-let-7i-5p, hsa-mir-21-5p, hsa-mir-155-5p, hsa-mir-24-3p, hsa-mir-146a-5p, hsa-mir-146b-5p, hsa-mir-221-3p, hsa-mir-222-3p, hsa-mir-34a-5p; downregulated—hsa-mir-194-5p, hsa-mir-139-3p, hsa-mir-874-3p, hsa-mir-139-5p) (Fig. 6A). We uncovered further 2 miRNAs that alter with full transdifferentiation, of which one was upregulated (hsa-mir-132-3p) and both strands of hsa-mir-150 (hsa-mir-150-3p and hsa-mir-150-5p) were downregulated (Fig. 6B). The data in early activation and perpetuation is also shown using Venn diagrams (Fig. 6C,D). Differentially expressed miRNAs that were correlated in both species seem to be controlling a number of genes during early initiation and perpetuation phase of HSC transdifferentiation (Fig. 6E,F). In particular we note potential for the identified commonly regulated miRNAs to extert significant control on HSC apoptosis; with linked apoptosis regulators including RelA, p53, TNF, AGO2, TIMP3, BTG2 and BACH2 (Fig. 6E,F). Since HSC apoptosis is a key mechanism for determining the balance between fibrosis progression and regression this represents an important finding that warrants future investigation.

Intriguingly, only miR-150 was significantly downregulated in fully activated HSC in both species, suggesting that expression of this miRNA may be crucial either for the phenotype of quiescent HSC or as an inhibitor of transdifferentiation.

miR-150 regulates HSC phenotype by controlling the expression of ZEB1, Collagen1A1 and αSMA.

**Figure 7:**
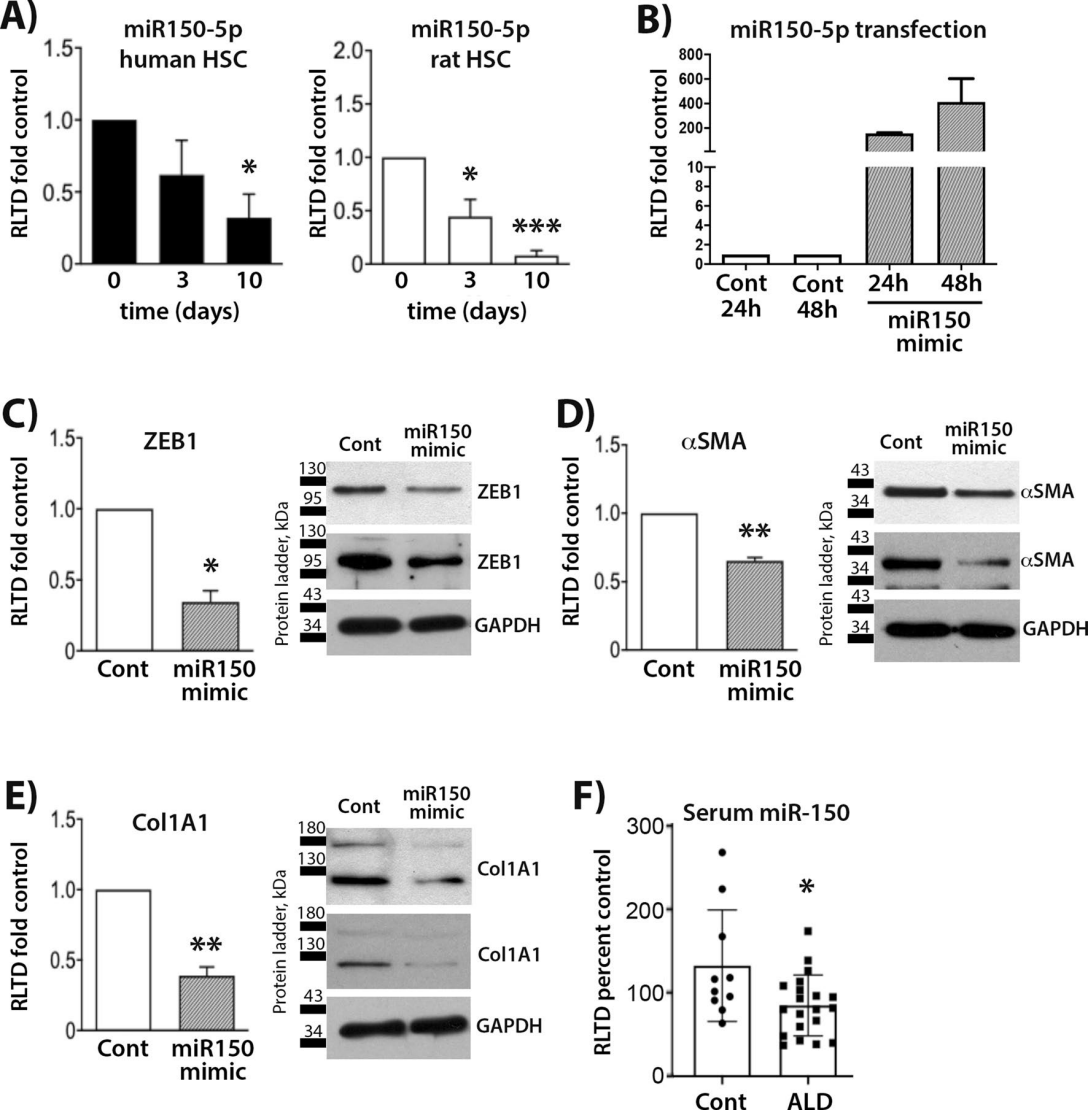
miR150 mimic decreases ZEB1, Col1A1 and αSMA expression in rat HSC. (**A**) miR150-5p expression levels in human HSC (left) and rat HSC (right) as quantified by qPCR (both n = 4). (**B**) miR150-5p expression levels in miR150 mimic transfected rat HSC as quantified by qPCR. Rat HSC were transfected with miR150 mimic (n = 3), expression levels of ZEB1 (**C**, left panel), αSMA (**D**, left panel) and Col1A1 (**E**, left panel) were quantified by qPCR and immunoblotted (**C**, right panel, **D**, right panel and **E**, right panel, respectively). (**F**) Serum of patients with advanced fibrosis due to ALD (n = 20) and healthy controls (n = 10) was collected and levels of miR150 quantified by qPCR. Statistical significance determined by Student’s t-test where * denotes *p* = 0.0168.

To validate the results of next generation sequencing and determine the potential for systematic miRNA expression analysis to identify novel regulators of HSC phenotype, we carried out follow-up studies on miR-150 which was identified as downregulated with transdifferentiation of both human and rat HSC. As shown in Fig. 7A, we were able to confirm a progressive diminution in expression of miR150 across day 0 to day 10, with a significant downregulation confirmed in both species by day 10 (Fig. 7A, left and right panel). We next assessed predicted targets of miR-150 (Supplementary Table 5). The gene list includes MYB, MUC4, EGR2, VEGFA, P2RX7, IGF2, ZEB1, CXCR4 and NOTCH3. We were particularly intrigued to see a number of transcription factors that may be affected by miR-150, such as ZEB1, EGR2 and MYB. In order to understand what impact the change of miR-150 expression might have on myofibroblast phenotype, we carried out transfection of activated rat HSC with a miR-150 mimic (Fig. 7B). Transfected HSC displayed a 200-fold increase in miR-150 levels 24 h post transfection, with levels further raising to 400-fold at 48 h. Transfected cells were harvested at 48 h and qPCR and western blot carried out for ZEB1, which is one of the putative targets of miR-150 (Fig. 7C). ZEB1 gene expression was significantly reduced in activated HSC transfected with miR-150 mimic, which was further reflected in reduced ZEB1 protein expression (Fig. 7C, right panel). We were next interested to know if increase in miR-150 affects the fibrogenic phenotype of activated HSC. Quantitative PCR and western blotting revealed a significant reduction in αSMA and Collagen1A1 (Col1A1) in HSC transfected with the miR-150 mimic (Fig. 7D,E). These data suggest that the expression of two major fibrogenic markers, αSMA and Col1A1, may be influenced downstream of changes in the expression of miR-150 during transdifferentiation of rodent and human HSC.

Hsa-miR-150 is decreased in serum of patients with advanced ALD.

Next, we were interested to assess whether circulating levels of miR-150 decrease in serum of patients with confirmed advanced fibrosis. To this end, we analysed 20 patients with clinical evidence of advanced fibrosis (grade 3–4) due to excessive alcohol consumption (ALD) and compared them to 10 healthy controls (Fig. 7F). Clinical characteristics of the ALD cohort are shown in Supplementary Table 6; characteristics of healthy control are in Supplementary Table 7. The data show evidence of significant miR-150 reduction in systemic circulation of ALD patients, suggesting it may offer a serum biomarker for detection of severe fibrosis in ALD.

## Discussion/conclusion

HSC transdifferentiation is an epigenetically regulated process involving remodelling of the DNA methylome, extensive changes in histone modifications and alterations in the expression of regulatory RNA species^12^. A detailed delineation of the epigenetic mechanisms controlling the profibrogenic phenotype of the HSC will advance understanding of the molecular control of liver fibrosis. MicroRNAs are epigenetic regulators that have shown considerable promise as therapeutic targets and biomarkers for a variety of human diseases^13,14^. In this study we employed a non-biased RNA-seq approach to document global changes in microRNA expression accompanying the initiation and perpetuation phases of HSC transdifferentiation. Employing a comparing approach between rat and human HSC, we identified a total of 15 microRNAs that display similar up- or downregulation in association with HSC transdifferentiation.

The strengths of our work were (i) the enhanced specificity and sensitivity of RNA-seq technology over microarrays for reporting differentially expressed transcripts, and (ii) a comparative analysis of real-time changes in microRNA expression between rodent and human HSC which allows greater confidence for identifying RNA regulators of fibrogenesis. An important limitation was use of the in vitro model HSC transdifferentiation which imperfectly recapitulates the molecular events occurring in situ within the diseased liver^15^. However, while “activated” HSC can be isolated from rodent liver, it is considerably more challenging to isolate HSC that are in the early initiation phase, indeed this would be exceptionally difficult from human liver.

Our discovery of just 15 microRNAs that are conserved with respect to their modulation in expression during HSC transdifferentiation clearly represents a minority of the total number of microRNAs we observed as modulated within each of species. Inherent variables in HSC biology may contribute to this discordance including HSC heterogeneity, differences in the kinetics of transdifferentiation between human and rat cells and/or distinct species-specific differences in the regulation of certain microRNAs. A number of technical variables are also important to highlight, in particular that while rat HSC are prepared from relatively young healthy adult males housed in highly controlled environments, by contrast human HSC are isolated from the “normal” margins of resected metastatic colorectal cancer tissue and from what are usually older (50 years+) patients of variable clinical status.

The initiation phase of transdifferentiation was associated with modulation of 15 microRNAs of which all but one, miR-874-3p, continued to display altered expression in the perpetuation phase. To date miR-874-3p is yet to be attributed a function in fibrosis, this likely reflecting the transient nature of its upregulation at initiation. Although miR-874 functions as a cell cycle suppressor in cancer^16,17^, we can only speculate what its transient increase may regulate in the early activating HSC. The other microRNAs (miR-194-5p, miR-139-5p and miR139-3p) that were upregulated during initiation remained increased in the fully activated HSC and have previously been implicated in various fibrogenesis related pathways. For example, miR-139 may form a regulatory axis with β-catenin to control Collagen I and αSMA levels in the bleomycin model of pulmonary fibrosis^18^. It may also function as a suppressor of HSC proliferation either through control of Rac1 or/and AKT1^10,19^. While there are as yet no reports of a specific function for the miR-139 class of microRNAs in HSC, miR-139-5p is observed to be downregulated in human non-alcoholic fatty liver disease (NAFLD) and primary billiary cholangitis (PBC)^20^ and over-expression of miR-139 in primary human fibroblasts blocks the stimulatory actions of TGFβ^21^ and inhibits epithelial-mesenchymal transition (EMT)^22^. Expression of miR150-5p was unchanged during initiation but instead was depressed in the perpetuation phase, this indicating it may exert negative control on functions of the myofibroblastic HSC. Indeed, over-expression of a miR-150-5p mimetic in activated HSC suppressed the pro-fibrogenic transcription factor ZEB1 which was previously shown to stimulate Collagen I and αSMA transcripts in the human LX-2 HSC cell line^23^. In our hands, expression of Collagen I and αSMA expression was inhibited by the mir-150 mimetic alongside loss of ZEB1. Of relevance to progression of liver fibrosis, miR-150 mimetic stimulated HSC apoptosis^24^ and by contrast Li and colleagues showed that over-expression of ZEB1 suppressed HSC apoptosis^23^. Hence, these observations suggest that a mir-150-ZEB1 axis exerts control over the fate and function of the activated HSC, this indicating that the therapeutic potential for mir-150 mimetics warrants further attention. In addition, we also observed that serum changes in miR-150 correlate with advanced human liver fibrosis, this further indicating relevance of the microRNA to the fibrogenic process.

Comparative analysis of rat and human HSC transdifferentiation revealed conserved upregulation of 9 microRNAs at the initiation phase with addition of miR-132-3p being selectively enhanced at the perpetuation phase. In the context of chronic kidney disease, miR-132 increases with pericyte to myofibroblast transdifferentiation and its antagomir is a suppressor of myofibroblast proliferation^25^. The function of miR-132 has to date not been studied in HSC or liver fibrosis. Of the 9 microRNAs induced at initiation, only miR221 and miR222 have been studied in HSC and are reported to control expression of the cell cycle inhibitors CDKN1B and CDKN1C^8^. While not yet studied in HSC, miR24-3p is a TGFβ regulator^26^, miR21-5p stimulates cardiac fibroblast proliferation^27^ and can target Smad7 to amplify TGFβ signalling; miR34a-5p targets LIN28A which has multiple functions including inhibition of miR biosynthesis and enhancement of p53-dependent senescence. The transcript miR-146a for which the -3p and -5p forms are upregulated at initiation is elevated in NAFLD^28^, while miR-139-5p is supressed in NAFLD and PBC^20^ and is able to inhibit EMT and c-Fos indicating a role in control of cell phenotype^20,22^. Our bioinformatics analysis highlighted miR-146a-5p as a potential immune regulator, showing functional associations with TNF, IL38G, IL17B, IL36RN, TLR10 and SFTP. Finally, the downregulated transcripts miR-340-5p, Let-7i-5p and miR-155-5p have no obvious links with phenotype transformation, fibrosis or liver disease and as such given their conserved regulation between rat and human HSC will be interesting to examine for novel functions in transdifferentiation.

In summary, by carrying out an unbiased RNAseq analysis of changes in microRNA expression and then asking which transcripts display conserved expression changes between rat and human HSC we have identified 15 microRNAs that have potential to functionally contribute to the HSC phenotype and fibrogenesis.

## Materials and methods

### Ethics

Authors hold appropriate licenses for animal experiments, which were issued/approved by local ethical committee and UK Home Office.

### Animals

Sprague Dawley male rats were purchased at around 12 weeks old and subsequently housed in RC2 cages. Environmental enrichment in form of chew sticks and cardboard tubes were provided. Rats had free access to water and RM3 diet (DBM diets, Broxburn, UK). Animals received humane care at all times and were maintained on a 12 h light/dark cycle. All procedures and experiments were approved by the Newcastle Animal Welfare and Ethical Review Board and performed under a UK Home Office licence. All methods were carried out in accordance with relevant guidelines and regulations.

### Human liver tissue

Human liver tissue was obtained from normal resection margin surrounding colorectal metastasis, with informed consent from adult patients undergoing surgical resection at the Freeman Hospital, Newcastle-upon-Tyne, UK. This study was approved by the Newcastle & North Tyneside Research Ethics Committee (REC reference 12/NE/0395). All methods were carried out in accordance with relevant guidelines and regulations.

### Hepatic stellate cell isolation and culture

Rat HSC were isolated from 250 g male Sprague-Dawleys and primary human HSC were isolated from normal tissue margins of surgically resected liver. Isolations were carried out by sequential perfusion with collagenase B (Roche) and pronase (Roche); quiescent HSC were separated by discontinuous density centrifugation in 11.5% Optiprep (Sigma Aldrich). HSC were seeded onto plastic (Corning), maintained at 37 °C (5% CO_2_) in Dulbecco's Modified Eagle's Media supplemented with 100 units/mL penicillin, 100 mg/mL streptomycin, 2 mM l-glutamine and 16% fetal calf serum. Cell cultures were maintained at 37 °C at an atmosphere of 5% CO_2_. Freshly isolated HSC (day 0) were considered quiescent and were cultured in plastic dishes to induce their transdifferentiation to fully activated HSC (day 10). HSC from both species were harvested at days 0, 1, 3, 5 and 10.

### Small RNA sequencing

Total RNA was isolated from cultured cells using Qiagen RNeasy mini kit. A total RNA input of 1 μg was used to prepare sample libraries with NEBNext Multiplex Small RNA Library Prep Set for Illumina. Briefly, total RNA was 3′ and 5′ adapter ligated. Index adapters and enrichment were performed by running a 15 cycle PCR reaction (as per manufacturer’s instructions). Small RNA fractions were purified using 6%TBE-PAGE gel electrophoresis and isolating the band corresponding to ~ 140 bp. Libraries were then purified and concentrated as dictated in the protocol. Library quality and quantity was assessed using a Bioanalyzer with a high-sensitivity DNA chip. All sample libraries were then diluted to a final concentration of 4 nM and sequenced for 36 cycles on an Illumina MiSeq using a V3 cartridge.

### Small RNA quantification and differential expression analysis

Raw fastq reads obtained from sample library sequencing were analysed using the Chimira pipeline^29^ (https://www.ebi.ac.uk/research/enright/software/chimira) to trim (adapter sequence “AGATCGGAAGAGC”), size selection, mapping and quality control analysis. MiRNA count data were normalized and corrected for bath effects, using DESeq2 package for R (v 3.01)^30^. Differential expression analysis was performed using DESeq2 package for R, MiRNAs with a log2 fold-change (logFC) of > 1 and an adjusted p value of < 0.05 were classified as differentially expressed. Further analysis was performed using a custom R script to generate volcano plot and heatmap matrices, gplots package for R (v 3.01) (https://CRAN.R-project.org/package=gplots).

### Ingenuity pathway analyses

MiRNAs with expression level that was considered significantly different were input into the Ingenuity Pathway Analysis tools (http://www.ingenuity.com) for functional annotation.

### SDS-PAGE and immunoblotting

Total cell lysates were generated using a lysate buffer (150 mM NaCl, 0.1% SDS, 1% Triton x-100, 0.5% Sodium Deoxycholic and 50 mM Tris, pH 8.0). Appropriate amount of protease and phosphatase inhibitors were added just before use. 4–12% Tris gel were poured and equivalent amount of lysate samples were loaded and electrophoresed. The run gels were transferred to nitrocellulose membrane and blocked for 1 h at room temperature in Tris-buffered saline containing 0.1% Tween-20 (TBS-T)/5% nonfat dry milk (Bio-Rad Laboratories). The membranes were then incubated with specific primary antibodies (ZEB1 (Santa Cruz Biotechnology, INC), COL1A1 (Santa Cruz Biotechnology, INC), α-SMA (Sigma Aldrich) and GAPDH (Abcam) overnight at 4^0^C. The blotted membranes were washed several times with TBS-T, then incubated with horseradish peroxidase-conjugated secondary antibodies for 1 h at RT. Positive proteins were visualized by enhanced chemiluminescence (ECL) and exposure to film (Kodak, UK).

### Transfection of miRNA-150 mimic into rat HSC

miR-150-5p mimic (5′-UCUCCCAACCCUUGUACCAGUG-3′) was synthesized by Qiagen, UK. Rat HSC were transfected 48 h after the isolation (day 2) with miR-150-5p mimic (10 nM) or with Silencer Select Pre-designed ZEB1 (Life Techologies) for 48 h using INTERFERin (Polyplus) according to the manufacturer’s protocol. A scramble negative control was purchased from Ambion. Cells were harvested and processed for isolation of total RNA and protein 48 h after transfection in order to verify the efficiency of the miRNA mimic.

### Control and ALD patient serum

The use of human blood for scientific research was approved by Newcastle and North Tyneside Local Research Ethics (approval number 10/H0906/41). All samples were collected subject to informed patient consent in writing. Serum was collected after centrifugation and miRNA isolation carried out using Qiagen miRNeasy Serum/Plasma Kit (Cat no. 217184, UK) starting from 200 μl of serum. Serum was spiked with miRNeasy serum/Plasma Spike-In Control (Qiagen Cat no. 219610, UK). Relative expression of miR-150 in serum and cells was measured using qPCR. Briefly, total miRNAs (50 ng) extracted from human serum or 100 ng of total RNA isolated from HSC cells were used to synthesize cDNA utilizing miScript Reverse Transcriptase Kit (Qiagen, UK) according to the manufacturer’s protocol and was then resuspended in 20 μl amounts of H2O. cDNA samples (1 μl) were used for qPCR in a total volume of 25 μl using the miScript SYBR Green PCR Kit (Qiagen, UK) and miScript Primer Assay (Qiagen, UK): Hs_miR-150-5p and Hs_RNU6-2. Quantitative PCR program: 15 min at 95 °C, then 40 cycles of 15 s at 94 °C, 30 s at 55 °C, 30 s at 70 °C, followed by ABI7500 machine predetermined melt curve. All reactions were normalized to the external control and expressed as relative level of transcriptional difference (RLTD).

### Statistical analysis

Results are shown as mean ± SD. Statistical comparisons were made using one-way analysis of variance. The statistical analysis was performed using GraphPad Prism software. *p* values < 0.05 were considered significant.

### Ethics approval and consent to participate

This study was approved by the Newcastle & North Tyneside Research Ethics Committee (REC reference 12/NE/0395). All consent forms are stored in Newcastle University Biobank.

## Supplementary Information

Supplementary information.

## Data Availability

The datasets generated during and/or analysed during the current study will be made available in appropriate database once the manuscript is accepted.

## Abbreviations

α-SMA: α-Smooth muscle actin
qHSC: Quiescent hepatic stellate cells
COL1a1: Collagen 1a1
SEM: Standard error of the mean
miR: Micro RNA
