# Supplementary material for: RNA sequencing reveals changes in the microRNAome of transdifferentiating hepatic stellate cells that are conserved between human and rat
Source: Sci Rep. 2020 Dec 10;10:21708. doi: 10.1038/s41598-020-78776-3 (PMC7728773; doi:10.1038/s41598-020-78776-3)

RNA sequencing reveals changes in the microRNAome of transdifferentiating hepatic stellate cells that are conserved between Human and Rat

Laura Sabater*[1], Luigi Locatelli*[1], Fiona Oakley[1], Timothy Hardy[1], Jeremy French[2], Stuart Robinson[2], Gourab Sen[2], Mann DA[§1], Mann J[§1]

[1] Newcastle Fibrosis Research Group, Bioscience Institute, Faculty of Medical Sciences, Newcastle University, Newcastle upon Tyne, UK.

[2] Department of Hepatobiliary Surgery, Newcastle upon Tyne Hospitals NHS Foundation Trust, Newcastle upon Tyne, UK.

Joint first authors*, joint senior authors[§]

Correspondence: Jelena Mann, Biosciences Institute, Faculty of Medical Sciences, 4th Floor, William Leech Building, Newcastle University, Framlington Place, Newcastle upon Tyne, NE2 4HH, UK. Tel +44 191 208 5902. E-mail Jelena.Mann@newcastle.ac.uk

Please find below all supplementary figures and tables as well as full western blot images.

Contents overview

Supplementary Figure 1	page 2
Supplementary Tables 1-7	pages 3-17
Whole images for western blots in Figure 6	page 18

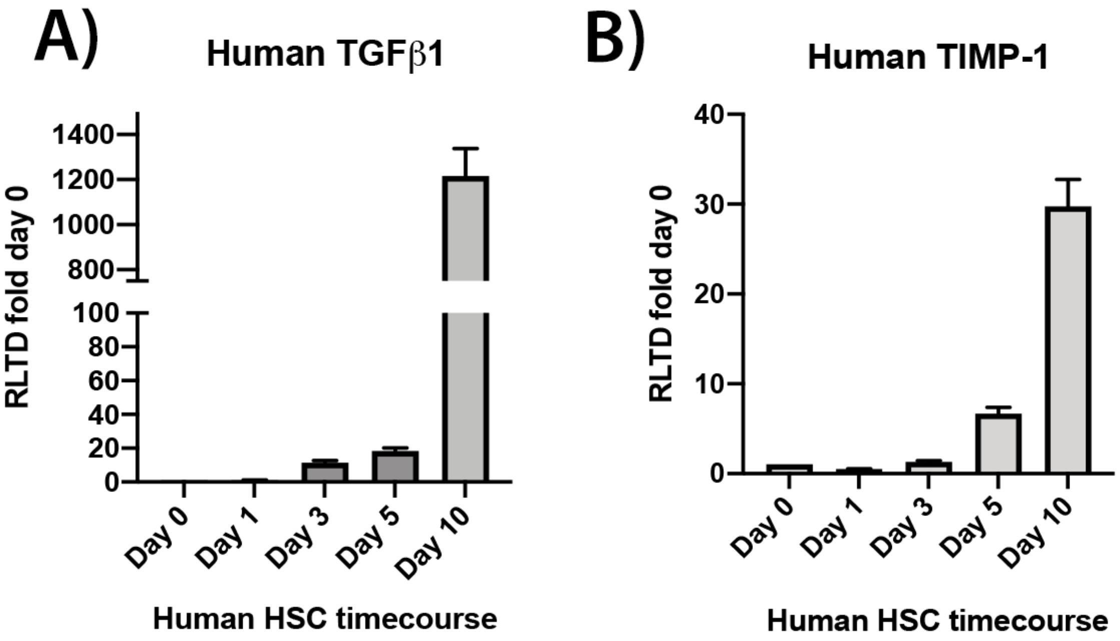

Supplementary Figure 1- Quantitative PCR carried out on time-course cDNA isolated from of human HSC preps used in the study showing in A) TGFβ1 and B) TIMP-1 transcript levels.

Supplementary Table 1 – Human HSC; early activation

miRNAs upregulated in early activation (day 0 to3)

miRNA	log2FoldChange	lfcSE	pvalue	padj
hsa-mir-4521	3.093665956	1.10842222	0.00525368	0.02777805
hsa-mir-98-5p	1.712408307	0.66729761	0.01028243	0.04683088
hsa-mir-501-3p	1.581409525	0.61738464	0.01042316	0.04700639
hsa-mir-511-5p	2.493714167	0.89861543	0.00551911	0.02884988
hsa-mir-34a-5p	2.296631888	0.7325486	0.00171781	0.01000241
hsa-mir-132-3p	2.77065521	0.76329665	0.00028358	0.0024157
hsa-mir-151a-5p	2.491799295	0.9617711	0.00957408	0.04448561
hsa-mir-148b-3p	1.383914077	0.54178156	0.01063782	0.04750869
hsa-mir-532-5p	1.581818503	0.42430286	0.00019297	0.00170708
hsa-mir-222-3p	2.214546926	0.53485399	3.47E-05	0.00038886
hsa-mir-221-3p	1.508757406	0.53221723	0.00458468	0.02452272
hsa-mir-146b-5p	2.291837685	0.65557037	0.00047239	0.00356906
hsa-let-7i-5p	1.680077045	0.38470528	1.26E-05	0.00018093
hsa-mir-24-3p	1.118726348	0.339062	0.00096866	0.00645774
hsa-mir-22-3p	2.441742349	0.48892328	5.91E-07	1.13E-05
hsa-mir-146a-5p	2.806629844	0.58278187	1.47E-06	2.70E-05
hsa-mir-155-5p	3.338306564	0.4251777	4.11E-15	6.30E-13
hsa-mir-21-5p	3.270012077	0.56503736	7.15E-09	2.19E-07
hsa-let-7g-5p	1.145965761	0.30629287	0.00018299	0.00165054
hsa-let-7f-5p	1.31837284	0.44570038	0.00309662	0.01695769

miRNAs downregulated in early activation (day 0 to 3)

miRNA	log2FoldChange	lfcSE	pvalue	padj
hsa-mir-27a-5p	-4.927912773	1.9402573	0.01109081	0.04905553
hsa-mir-3938	-4.580566318	1.7619624	0.00933064	0.04432434
hsa-mir-5585-3p	-4.943326329	1.81330514	0.00640795	0.03239185
hsa-mir-1273g-3p	-2.921192717	0.68967871	2.28E-05	0.00028607
hsa-mir-466	-5.670071825	1.7465658	0.0011687	0.00746667
hsa-mir-3916	-5.13863224	1.98851907	0.00976192	0.04490485
hsa-mir-1972	-5.052428399	1.67827265	0.00260824	0.01445529
hsa-mir-433-3p	-4.894555857	1.88316804	0.00934665	0.04432434
hsa-mir-6516-3p	-5.259789355	1.66248781	0.0015572	0.00930278
hsa-mir-1285-3p	-4.618994712	1.5261208	0.00247301	0.01387298
hsa-mir-1290	-4.745242374	1.74408883	0.00651326	0.03256628
hsa-mir-5708	-4.881029375	1.57129356	0.00189395	0.01089024
hsa-mir-3135b	-4.802068811	1.4320667	0.0007987	0.00556672
hsa-mir-3135a	-5.956721432	1.54582873	0.00011648	0.00107163
hsa-mir-4512	-5.890172877	1.64089444	0.00033117	0.00267261
hsa-mir-6073	-5.651257456	1.56814753	0.00031362	0.00257619

hsa-mir-134-5p	-3.946789236	1.50197269	0.00859549	0.04162029
hsa-mir-1244	-4.637643221	1.69109818	0.00609947	0.03152535
hsa-mir-4485-5p	-6.26026085	1.81728182	0.00057138	0.00423927
hsa-mir-1254	-3.865340287	1.08759483	0.00037938	0.00300889
hsa-mir-6131	-3.115724024	1.02750734	0.00242687	0.01378224
hsa-mir-663b	-5.622493849	1.44316614	9.78E-05	0.00093741
hsa-mir-5684	-6.427333807	1.55132326	3.43E-05	0.00038886
hsa-mir-1268a	-3.456401133	1.02722442	0.000766	0.00542093
hsa-mir-4516	-5.627277501	1.44323689	9.66E-05	0.00093741
hsa-mir-3665	-4.242007579	1.21358478	0.00047329	0.00356906
hsa-mir-4466	-6.595338744	1.64486523	6.08E-05	0.00063572
hsa-mir-620	-4.364035891	1.30489104	0.0008247	0.00566214
hsa-mir-3196	-6.728749894	1.58324694	2.14E-05	0.00028099
hsa-mir-1273h-5p	-3.869696321	1.18461195	0.00108833	0.00705118
hsa-mir-194-5p	-2.096201418	0.80803176	0.00948095	0.04448561
hsa-mir-503-5p	-3.3685338	1.07217238	0.00167922	0.00990309
hsa-mir-3656	-3.653776641	1.1302531	0.00122623	0.00772696
hsa-mir-3609	-4.296809631	1.25632415	0.00062588	0.00456992
hsa-mir-1273c	-4.61190671	1.15364337	6.40E-05	0.00065385
hsa-mir-4459	-3.898721265	0.89661574	1.37E-05	0.00019128
hsa-mir-3651	-3.531860415	0.91322927	0.00010998	0.0010325
hsa-mir-1324	-2.622034726	0.79592968	0.00098666	0.00648374
hsa-mir-3159	-4.663938815	1.09704913	2.12E-05	0.00028099
hsa-mir-8065	-4.830925554	1.174602	3.91E-05	0.00041813
hsa-mir-3687	-5.779282903	1.20915367	1.76E-06	3.11E-05
hsa-mir-3182	-2.523345367	0.79210733	0.00144449	0.00885954
hsa-mir-3195	-2.980999403	1.11707862	0.00761755	0.03767822
hsa-mir-3648	-4.915717868	0.93291271	1.37E-07	3.00E-06
hsa-mir-5095	-4.474421847	0.96064893	3.20E-06	5.07E-05
hsa-mir-663a	-4.147654839	1.00321237	3.56E-05	0.00038982
hsa-mir-1273d	-4.937200371	0.85938643	9.19E-09	2.64E-07
hsa-mir-4508	-3.066511607	0.95661186	0.00134783	0.00837839
hsa-mir-3929	-4.363879095	0.75077366	6.15E-09	2.02E-07
hsa-mir-1273f	-4.561024318	0.96184835	2.12E-06	3.48E-05
hsa-mir-1248	-5.844167904	1.22654183	1.89E-06	3.22E-05
hsa-mir-566	-4.968170163	0.91999426	6.66E-08	1.80E-06
hsa-mir-139-3p	-3.796008397	0.89666258	2.30E-05	0.00028607
hsa-mir-7851-3p	-5.534751787	1.03940847	1.01E-07	2.45E-06
hsa-mir-874-3p	-2.904417211	0.69983333	3.32E-05	0.00038886
hsa-mir-4472	-5.907184194	0.92020216	1.37E-10	6.29E-09
hsa-mir-214-3p	-2.816678459	0.88824739	0.00151888	0.00919322
hsa-mir-7704	-1.791531369	0.62740765	0.00429758	0.02325752
hsa-mir-370-3p	-4.245796718	1.07999957	8.45E-05	0.0008449

hsa-mir-486-5p	-4.283353015	0.66092582	9.12E-11	4.66E-09
hsa-mir-1273e	-4.609534244	0.68933721	2.28E-11	1.75E-09
hsa-mir-3908	-5.587417263	1.04395552	8.69E-08	2.22E-06
hsa-mir-4488	-7.173070799	1.0315383	3.56E-12	3.27E-10
hsa-mir-127-3p	-2.962777458	1.08440502	0.00629189	0.03215856
hsa-mir-8485	-3.752937319	0.74304056	4.40E-07	8.80E-06
hsa-mir-4792	-3.643587548	1.07410788	0.00069334	0.00498339
hsa-mir-619-5p	-5.689610026	0.90884548	3.84E-10	1.61E-08
hsa-mir-4419a	-3.139784959	0.68898735	5.19E-06	7.95E-05
hsa-mir-1285-5p	-5.213091064	0.8922125	5.13E-09	1.82E-07
hsa-mir-4485-3p	-6.390877291	1.07848854	3.11E-09	1.19E-07
hsa-mir-5588-5p	-5.962099902	0.91678455	7.86E-11	4.52E-09
hsa-mir-6087	-3.333967067	0.92053131	0.00029258	0.002447
hsa-mir-8086	-5.905112165	0.90650923	7.31E-11	4.52E-09
hsa-mir-1303	-2.14617108	0.64512565	0.00087866	0.00594384
hsa-mir-5096	-5.253503137	0.71995622	2.94E-13	3.38E-11
hsa-mir-3960	-6.43711702	0.77886407	1.40E-16	3.22E-14
hsa-mir-7110-3p	-7.391385067	0.72973737	4.12E-24	1.89E-21
hsa-mir-1273g-5p	-5.039410363	1.89525308	0.00783813	0.03835679
hsa-mir-139-5p	-3.260452817	0.87755008	0.00020288	0.00176084
hsa-mir-1273a	-3.929675481	0.73932009	1.07E-07	2.45E-06
hsa-mir-7641	-4.0301839	0.90364609	8.20E-06	0.00012167
hsa-mir-1246	-2.556101169	0.61554299	3.29E-05	0.00038886
hsa-mir-4791	-3.54812285	0.67617777	1.54E-07	3.23E-06
hsa-mir-320a	-2.411148014	0.68363384	0.00042036	0.00327741

Supplementary Table 2 – Human HSC

miRNAs upregulated during transdifferentiation

miRNA	log2FoldChange	lfcSE	pvalue	padj
hsa-mir-340-3p	3.601571086	1.31776359	0.00627425	0.02475164
hsa-mir-133a-3p	3.441861055	1.21704088	0.00468315	0.01936301
hsa-mir-340-5p	2.619855145	1.02135308	0.01031512	0.03791027
hsa-mir-4521	3.049608677	1.14911491	0.0079573	0.03001439
hsa-mir-21-3p	2.155375494	0.74605405	0.00386427	0.01661638
hsa-mir-98-5p	2.009084435	0.68512191	0.00336301	0.01475607
hsa-mir-378d	2.449568459	0.87348635	0.00504169	0.02045213
hsa-mir-132-5p	3.65147776	1.03739749	0.0004318	0.00241135
hsa-mir-378a-5p	4.271339771	1.19553896	0.00035328	0.00201273
hsa-mir-34a-5p	3.760660708	0.76602358	9.14E-07	1.19E-05
hsa-mir-1249-3p	2.745379447	0.95411971	0.00400973	0.01707113

hsa-mir-132-3p	3.260912844	0.80067194	4.65E-05	0.00036329
hsa-mir-128-3p	1.816963439	0.65171054	0.00530359	0.02131351
hsa-mir-148b-3p	2.61547283	0.56985875	4.44E-06	4.34E-05
hsa-mir-100-5p	5.165626523	0.95317187	5.98E-08	1.19E-06
hsa-mir-27a-3p	1.257499156	0.50601038	0.01295059	0.04559109
hsa-mir-99b-5p	1.759420475	0.59302363	0.00300854	0.01371143
hsa-mir-532-5p	1.228350116	0.45160567	0.00652895	0.02525348
hsa-mir-23a-3p	1.736766828	0.52158204	0.00086907	0.00461356
hsa-mir-222-3p	2.754278638	0.57106608	1.41E-06	1.52E-05
hsa-mir-221-3p	2.474904608	0.57049053	1.44E-05	0.00012606
hsa-mir-146b-5p	2.776877912	0.70505605	8.20E-05	0.00056861
hsa-mir-191-5p	1.105065215	0.43500972	0.01107501	0.0403581
hsa-let-7f-5p	1.285417013	0.48002429	0.00741038	0.02819879
hsa-mir-24-3p	1.969307213	0.36375205	6.17E-08	1.19E-06
hsa-mir-22-3p	2.924146451	0.5257828	2.67E-08	6.05E-07
hsa-mir-146a-5p	3.357794276	0.6273702	8.69E-08	1.49E-06
hsa-mir-155-5p	3.056418175	0.45668747	2.19E-11	1.05E-09
hsa-mir-378a-3p	2.618769456	0.67416496	0.00010256	0.00067845
hsa-mir-21-5p	4.648869207	0.60865198	2.21E-14	3.16E-12
hsa-let-7g-5p	1.017066526	0.33025369	0.00207242	0.01024302
hsa-let-7i-5p	1.793266087	0.41516224	1.56E-05	0.00013189

miRNAs downregulated during transdifferentiation

miRNA	log2FoldChange	lfcSE	pvalue	padj
hsa-mir-451a	-6.629754956	2.71178354	0.01449345	0.04985746
hsa-mir-1273g-5p	-4.991088809	1.92495256	0.00951878	0.03528513
hsa-mir-3916	-6.318695226	2.15943794	0.00343259	0.01490923
hsa-mir-1972	-5.482435832	1.72017417	0.00143687	0.00726888
hsa-mir-3149	-3.874221701	1.56052311	0.01304117	0.04559109
hsa-mir-3658	-5.098676505	1.71132585	0.0028884	0.01335496
hsa-mir-3133	-4.471722135	1.80830453	0.01340287	0.0464777
hsa-mir-6516-3p	-4.885476	1.62319929	0.00261435	0.0124908
hsa-mir-1285-3p	-4.358047812	1.42338505	0.00220048	0.01075234
hsa-mir-1290	-4.653685435	1.71248571	0.00657765	0.02525348
hsa-mir-5708	-4.314091766	1.45570237	0.00304082	0.01371143
hsa-mir-1302	-5.444568539	1.94138785	0.0050399	0.02045213
hsa-mir-23a-5p	-3.219848336	1.17295637	0.00604978	0.0240871
hsa-mir-150-3p	-4.979144741	1.37344253	0.00028862	0.00172372
hsa-mir-4532	-5.658028751	1.74779296	0.00120691	0.00617823
hsa-mir-3135b	-3.441374751	1.21230625	0.00452976	0.01891065
hsa-mir-3135a	-5.944541996	1.51775312	8.98E-05	0.00060321
hsa-mir-4512	-4.473741561	1.51061882	0.00306116	0.01371143
hsa-mir-6073	-6.735399212	1.69379047	6.99E-05	0.00049295

hsa-mir-4492	-6.794255107	1.57078856	1.52E-05	0.00013095
hsa-mir-1244	-4.266073568	1.69031871	0.01160847	0.04159703
hsa-mir-4485-5p	-6.772787632	1.89245153	0.00034512	0.00200542
hsa-mir-1254	-4.15697675	1.09449851	0.00014583	0.00092855
hsa-mir-6131	-4.38292473	1.11617811	8.61E-05	0.00058774
hsa-mir-663b	-5.310749105	1.31581316	5.43E-05	0.00041731
hsa-mir-320c	-2.621218215	1.00796179	0.00930844	0.03480546
hsa-mir-5684	-5.301871404	1.42071128	0.00019008	0.00116766
hsa-mir-1268a	-4.313652122	1.08203058	6.70E-05	0.00048223
hsa-mir-4516	-8.463199998	1.6352076	2.27E-07	3.49E-06
hsa-mir-3665	-6.141942622	1.38469456	9.18E-06	8.58E-05
hsa-mir-4466	-7.672762293	1.77824865	1.60E-05	0.00013211
hsa-mir-620	-3.606718178	1.25533976	0.00406464	0.01713524
hsa-mir-3196	-7.089551839	1.60780444	1.04E-05	9.30E-05
hsa-mir-1273h-5p	-4.237081508	1.2280943	0.00056032	0.00308437
hsa-mir-194-5p	-2.513186138	0.84004186	0.00277393	0.0130481
hsa-mir-3656	-7.487343625	1.55156194	1.40E-06	1.52E-05
hsa-mir-3609	-4.759189383	1.30804883	0.00027435	0.00166155
hsa-mir-1273c	-4.275876387	1.12631199	0.00014684	0.00092855
hsa-mir-4459	-4.497945238	0.93005333	1.32E-06	1.50E-05
hsa-mir-3651	-3.241159869	0.90765559	0.00035574	0.00201273
hsa-mir-1324	-2.803661088	0.81334173	0.00056666	0.00308437
hsa-mir-3159	-3.805802599	1.06063718	0.00033294	0.00196115
hsa-mir-8065	-5.39277989	1.22310679	1.04E-05	9.30E-05
hsa-mir-4497	-3.738443859	1.13587627	0.00099746	0.00523058
hsa-mir-3687	-5.789616706	1.18674687	1.07E-06	1.28E-05
hsa-mir-4443	-1.696225943	0.67110968	0.01148797	0.04151114
hsa-mir-3182	-2.051684651	0.82048503	0.01239918	0.04406319
hsa-mir-3195	-6.357976512	1.34215125	2.17E-06	2.27E-05
hsa-mir-3648	-4.983272207	0.92898671	8.13E-08	1.46E-06
hsa-mir-5095	-4.897751149	1.00148299	1.01E-06	1.24E-05
hsa-mir-223-3p	-2.950505524	0.94377577	0.00177034	0.0088517
hsa-mir-663a	-4.16796229	1.04573527	6.73E-05	0.00048223
hsa-mir-1273d	-5.134668888	0.86038776	2.40E-09	6.89E-08
hsa-mir-4508	-4.915802905	1.06285399	3.74E-06	3.74E-05
hsa-mir-3929	-4.703097195	0.76670472	8.56E-10	3.07E-08
hsa-mir-1273f	-5.621943787	1.03946645	6.36E-08	1.19E-06
hsa-mir-1275	-2.684751081	0.79698078	0.00075537	0.0040601
hsa-mir-1248	-6.931573082	1.32420684	1.65E-07	2.63E-06
hsa-mir-566	-6.266096117	1.00761819	5.01E-10	1.96E-08
hsa-mir-139-3p	-3.764457324	0.93712661	5.89E-05	0.00043696
hsa-mir-7851-3p	-5.642842513	1.06314908	1.11E-07	1.84E-06

hsa-mir-874-3p	-1.961424132	0.71859767	0.00634273	0.02479431
hsa-mir-4472	-5.260226746	0.86722226	1.31E-09	4.35E-08
hsa-mir-7704	-2.761206329	0.67528152	4.33E-05	0.00034503
hsa-mir-486-5p	-4.696591722	0.68670393	7.96E-12	4.89E-10
hsa-mir-1273e	-5.394096141	0.73140402	1.64E-13	1.77E-11
hsa-mir-3908	-5.419655604	1.07813176	4.98E-07	6.70E-06
hsa-mir-4488	-10.05682518	1.37828509	2.95E-13	2.54E-11
hsa-mir-8485	-3.833456212	0.78243891	9.61E-07	1.22E-05
hsa-mir-4792	-4.366253949	1.1571984	0.00016122	0.00100468
hsa-mir-619-5p	-5.09315768	0.91454721	2.56E-08	6.05E-07
hsa-mir-4419a	-4.193381492	0.74533016	1.84E-08	4.66E-07
hsa-mir-1285-5p	-5.632494665	0.94334189	2.36E-09	6.89E-08
hsa-mir-106b-3p	-1.743361255	0.53670843	0.00116119	0.00601579
hsa-mir-4485-3p	-6.711689265	1.13815177	3.70E-09	9.95E-08
hsa-mir-5588-5p	-6.424129887	0.97303578	4.05E-11	1.74E-09
hsa-mir-6087	-5.112499245	1.00266964	3.42E-07	4.90E-06
hsa-mir-8086	-6.49444848	0.9685076	2.01E-11	1.05E-09
hsa-mir-1303	-2.673185531	0.69466084	0.00011899	0.00077527
hsa-mir-5096	-5.553304694	0.76827918	4.89E-13	3.51E-11
hsa-mir-3960	-9.20842295	0.93829785	9.80E-23	2.11E-20
hsa-mir-7110-3p	-8.343799603	0.78430473	1.97E-26	8.49E-24
hsa-let-7c-5p	-2.601618188	0.85874501	0.00244912	0.0118328
hsa-mir-1273g-3p	-3.489203732	0.74406565	2.74E-06	2.81E-05
hsa-mir-150-5p	-2.406259991	0.53280176	6.29E-06	6.02E-05
hsa-mir-139-5p	-2.779974447	0.94506343	0.00326548	0.01447585
hsa-mir-1273a	-4.079851735	0.79659769	3.03E-07	4.49E-06
hsa-mir-7641	-4.740840667	0.97570317	1.18E-06	1.37E-05
hsa-mir-1246	-2.673694045	0.66393243	5.65E-05	0.00042607
hsa-mir-4791	-4.038416091	0.72985478	3.14E-08	6.76E-07
hsa-mir-320a	-3.029366244	0.73822413	4.07E-05	0.00033004
hsa-mir-122-5p	-5.406460563	1.07048877	4.41E-07	6.11E-06
hsa-let-7b-5p	-1.346829069	0.45047602	0.00279169	0.0130481

Supplementary Table 3 – Rat HSC; early activation

miRNAs upregulated in early activation (day 0 to 3)

miRNA	log2FoldChange	lfcSE	pvalue	padj
rno-mir-142-3p	3.556145632	1.11242457	0.00138984	0.00678148
rno-mir-18a-3p	3.071852997	0.99473146	0.00201425	0.00950055
rno-mir-221-5p	2.416554071	0.95516627	0.01140682	0.04362337
rno-mir-29b-3p	4.366207502	1.19455553	0.00025709	0.00161682
rno-mir-330-3p	2.395137907	0.8978615	0.00763945	0.03060809

rno-mir-147	2.277161938	0.77180634	0.0031733	0.01425467
rno-mir-877	2.642185985	0.65831926	5.98E-05	0.00043405
rno-mir-24-2-5p	1.489974273	0.50708402	0.00329998	0.0145921
rno-mir-125b-1-3p	3.590641396	1.16562147	0.00206686	0.00958889
rno-mir-20a-5p	1.38664249	0.56164263	0.01355271	0.0498106
rno-mir-99b-3p	1.820009414	0.39954453	5.23E-06	4.36E-05
rno-mir-185-5p	1.492254981	0.41635008	0.0003382	0.00199395
rno-mir-17-5p	2.117253674	0.58094406	0.00026791	0.00164821
rno-mir-34a-5p	3.91919794	0.56733386	4.91E-12	1.07E-10
rno-mir-1839-5p	1.955191932	0.39541761	7.63E-07	6.96E-06
rno-mir-466b-5p	1.241536118	0.33024679	0.00017031	0.00112033
rno-mir-181b-5p	2.327619409	0.27277519	1.42E-17	5.04E-16
rno-mir-222-3p	3.642152891	0.47725873	2.32E-14	6.57E-13
rno-mir-155-5p	5.008190197	0.50785845	6.12E-23	2.89E-21
rno-mir-466c-5p	1.649265034	0.30768494	8.31E-08	9.41E-07
rno-mir-221-3p	2.388146496	0.47037399	3.83E-07	3.74E-06
rno-mir-146b-5p	4.137153652	0.36524025	9.62E-30	1.36E-27
rno-mir-24-3p	1.253626323	0.23464355	9.16E-08	9.97E-07
rno-mir-21-5p	1.622988083	0.30850635	1.43E-07	1.50E-06
rno-mir-146a-5p	1.796355245	0.32983278	5.14E-08	6.62E-07
rno-let-7i-5p	2.125127562	0.20832843	1.97E-24	1.11E-22

miRNAs downregulated in early activation (day 0 to 3)

miRNA	log2FoldChange	lfcSE	pvalue	padj
rno-mir-497-5p	-3.938305767	1.32953189	0.00305474	0.01394343
rno-mir-150-3p	-3.830748017	1.34870014	0.00450675	0.01932438
rno-mir-3559-3p	-3.889959012	1.09995108	0.00040549	0.00234192
rno-mir-192-3p	-3.436412505	1.27326077	0.00695676	0.0289524
rno-mir-126a-5p	-5.2844019	1.11098752	1.97E-06	1.69E-05
rno-mir-195-3p	-4.106314218	1.08315616	0.00015	0.00103539
rno-mir-874-3p	-2.904655526	0.86485226	0.00078351	0.00418363
rno-mir-200a-3p	-1.972109602	0.70752809	0.00531454	0.02244799
rno-mir-194-3p	-6.248034956	1.04296216	2.09E-09	2.96E-08
rno-mir-10b-5p	-1.858796989	0.6972586	0.00767906	0.03060809
rno-mir-375-3p	-5.365167349	0.98883741	5.77E-08	7.10E-07
rno-mir-139-3p	-4.156017551	0.81266825	3.15E-07	3.19E-06
rno-mir-199a-5p	-4.554112308	1.17773869	0.00011026	0.00078009
rno-mir-322-3p	-2.781796435	0.73976903	0.00016967	0.00112033
rno-mir-1843a-3p	-1.746201357	0.54453014	0.00134218	0.00666383
rno-mir-125b-2-3p	-2.083433696	0.59977003	0.00051329	0.00284828
rno-mir-145-5p	-2.371464996	0.91983312	0.00993317	0.03904289
rno-mir-101b-3p	-1.695373079	0.59687418	0.00450542	0.01932438
rno-mir-194-5p	-4.810992367	0.70671866	9.93E-12	2.01E-10

rno-mir-122-3p	-5.00391986	0.80710434	5.65E-10	8.42E-09
rno-mir-200b-3p	-3.168003554	0.47046122	1.65E-11	3.12E-10
rno-mir-505-3p	-1.50680401	0.42733995	0.00042187	0.00238781
rno-mir-455-3p	-4.097011193	0.64418898	2.02E-10	3.17E-09
rno-mir-30e-5p	-1.032882149	0.32009411	0.0012518	0.00632604
rno-mir-30c-2-3p	-1.43028656	0.4327786	0.00095013	0.00488885
rno-mir-100-5p	-2.514896091	0.59892156	2.68E-05	0.00021072
rno-mir-26b-5p	-1.804649082	0.33610409	7.90E-08	9.32E-07
rno-mir-148a-3p	-2.01528925	0.40010279	4.73E-07	4.46E-06
rno-mir-31a-5p	-2.174310441	0.59867803	0.00028139	0.00169435
rno-mir-199a-3p	-3.053475666	0.75737457	5.54E-05	0.00041247
rno-mir-150-5p	-2.484915536	0.44129667	1.79E-08	2.42E-07
rno-mir-29a-3p	-1.188716242	0.35960418	0.00094765	0.00488885
rno-mir-23b-3p	-3.686734222	0.43154486	1.31E-17	5.04E-16
rno-mir-192-5p	-6.426735128	0.61950554	3.26E-25	2.30E-23
rno-mir-10a-5p	-3.099392857	0.39785762	6.69E-15	2.10E-13
rno-let-7d-3p	-1.059175361	0.30978218	0.00062829	0.00341936
rno-mir-139-5p	-3.436204005	0.45312433	3.37E-14	8.66E-13
rno-mir-27b-3p	-2.819929586	0.37876345	9.69E-14	2.28E-12
rno-mir-126a-3p	-7.146214151	0.49421219	2.17E-47	6.15E-45
rno-mir-99a-5p	-1.902442857	0.29205813	7.32E-11	1.22E-09
rno-let-7b-5p	-1.742634794	0.40041057	1.35E-05	0.00010902
rno-let-7c-5p	-1.379356592	0.32988378	2.90E-05	0.00022165
rno-mir-26a-5p	-2.290980833	0.21919238	1.44E-25	1.35E-23
rno-mir-125b-5p	-1.698022386	0.45234952	0.00017418	0.00112033
rno-mir-122-5p	-5.181809071	0.77471924	2.25E-11	3.98E-10

Supplementary Table 4 – Rat HSC

miRNAs upregulated during transdifferentiation

miRNA	log2FoldChange	lfcSE	pvalue	padj
rno-mir-344a-3p	3.793983515	1.320604	0.00406703	0.01646378
rno-mir-224-5p	3.413848783	1.28043455	0.00767222	0.0271763
rno-mir-149-5p	3.134069163	1.0027914	0.00177597	0.00855957
rno-mir-504	5.328054699	1.88762841	0.00476332	0.01867223
rno-mir-212-3p	3.384961347	1.37143755	0.01358002	0.04436139
rno-mir-183-5p	2.414428202	0.99337087	0.01507632	0.04817867
rno-mir-18a-3p	2.691192454	0.98408405	0.00624337	0.02353271
rno-mir-221-5p	2.350031542	0.93214466	0.0116988	0.03864548
rno-mir-760-3p	3.445816119	1.02704293	0.00079341	0.00424112
rno-mir-296-3p	5.779121389	1.33430888	1.48E-05	0.00011785

rno-mir-29b-3p	3.237991308	1.19885373	0.00691509	0.02541294
rno-mir-298-5p	6.310416854	1.33647874	2.34E-06	2.02E-05
rno-mir-132-3p	3.139088834	0.8770025	0.00034446	0.00191081
rno-mir-218a-5p	2.163696839	0.78192083	0.00565477	0.02159094
rno-mir-702-3p	3.342008364	0.85291368	8.92E-05	0.00055772
rno-mir-877	2.361853956	0.64858028	0.00027097	0.00156209
rno-mir-3102	4.215539873	0.91741997	4.33E-06	3.64E-05
rno-mir-24-2-5p	1.305474203	0.49436672	0.00827349	0.02895723
rno-mir-484	1.784071659	0.56633838	0.00163166	0.00799514
rno-mir-615	6.58352348	1.19520395	3.62E-08	3.55E-07
rno-mir-145-5p	1.963672391	0.75088265	0.00891877	0.03048975
rno-mir-324-3p	1.163462499	0.38748652	0.00267694	0.01210802
rno-mir-214-3p	2.85062594	0.70133203	4.81E-05	0.00032151
rno-mir-125b-1-3p	7.593472154	1.10139784	5.41E-12	9.94E-11
rno-mir-99b-3p	2.349106763	0.38289359	8.51E-10	1.14E-08
rno-mir-185-5p	2.314610464	0.39719859	5.63E-09	6.90E-08
rno-mir-17-5p	1.513768055	0.57861379	0.00889151	0.03048975
rno-mir-34a-5p	4.028363097	0.56115884	7.04E-13	1.48E-11
rno-mir-1839-5p	2.184304872	0.38630342	1.56E-08	1.77E-07
rno-mir-210-3p	2.743857031	0.47951387	1.05E-08	1.24E-07
rno-mir-1249	1.531838788	0.45859251	0.00083686	0.00431646
rno-mir-31a-5p	1.496485173	0.55806552	0.00732802	0.02659799
rno-mir-199a-3p	1.925243515	0.68644647	0.00503709	0.01948559
rno-mir-181b-5p	1.868422591	0.27078284	5.20E-12	9.94E-11
rno-mir-143-3p	3.06722427	0.72068213	2.08E-05	0.00015296
rno-mir-222-3p	4.099500485	0.47396367	5.18E-18	1.52E-16
rno-mir-155-5p	2.854106981	0.51178647	2.45E-08	2.57E-07
rno-mir-221-3p	3.118809262	0.46736749	2.50E-11	3.87E-10
rno-mir-146b-5p	3.636591273	0.36429637	1.82E-23	1.07E-21
rno-let-7e-5p	1.705317443	0.19332102	1.13E-18	3.70E-17
rno-mir-24-3p	1.379986885	0.2332727	3.30E-09	4.22E-08
rno-mir-21-5p	1.521790683	0.30789824	7.71E-07	6.87E-06
rno-mir-146a-5p	1.424975816	0.32952676	1.53E-05	0.00011838
rno-let-7i-5p	1.936527401	0.20794617	1.25E-20	4.58E-19

miRNAs downregulated during transdifferentiation

miRNA	log2FoldChange	lfcSE	pvalue	padj
rno-mir-335	-4.498857247	1.30528787	0.0005676	0.00309029
rno-mir-6215	-3.839781351	1.32774348	0.00382846	0.01607955
rno-mir-6320	-2.775544257	1.14568882	0.01540994	0.04819704
rno-mir-203a-3p	-3.482850777	1.12383673	0.00194128	0.00920542
rno-mir-497-5p	-3.095947885	1.2164429	0.01092518	0.03650004

rno-mir-150-3p	-5.100472916	1.35737198	0.00017154	0.00100868
rno-mir-3559-3p	-3.248982272	0.96994322	0.0008091	0.00424778
rno-mir-192-3p	-5.943394771	1.39437937	2.02E-05	0.00015246
rno-mir-126a-5p	-5.963238724	1.11098752	7.98E-08	7.57E-07
rno-mir-195-3p	-4.425752823	1.08395761	4.45E-05	0.00030402
rno-mir-148a-5p	-2.326531482	0.73383203	0.00152236	0.007586
rno-mir-30b-3p	-1.722478232	0.66769273	0.00988728	0.03341217
rno-mir-200a-3p	-3.210230727	0.77354594	3.32E-05	0.00023839
rno-mir-194-3p	-6.92687182	1.04296216	3.10E-11	4.56E-10
rno-mir-30e-3p	-1.631666969	0.55202096	0.00311853	0.01348306
rno-mir-375-3p	-7.132135957	1.06081491	1.78E-11	2.90E-10
rno-mir-139-3p	-5.129640904	0.90914607	1.68E-08	1.83E-07
rno-mir-1843a-3p	-1.470064507	0.49973843	0.0032645	0.0139096
rno-mir-101b-3p	-1.646214528	0.57333531	0.00408794	0.01646378
rno-mir-194-5p	-5.345133771	0.700049	2.25E-14	5.09E-13
rno-mir-122-3p	-8.84798885	1.11370638	1.95E-15	4.77E-14
rno-mir-532-3p	-1.39213834	0.4889499	0.00441056	0.01752303
rno-mir-320-5p	-1.379656022	0.47817391	0.00391091	0.01619446
rno-mir-200b-3p	-4.019265169	0.48113859	6.62E-17	1.77E-15
rno-mir-505-3p	-1.706336957	0.41292118	3.59E-05	0.00025135
rno-mir-455-3p	-1.981187129	0.5466664	0.00028994	0.00163929
rno-mir-30c-2-3p	-1.663518148	0.4220059	8.08E-05	0.0005166
rno-mir-30a-3p	-1.447351954	0.3729392	0.00010406	0.00063735
rno-mir-26b-5p	-2.276257573	0.33119769	6.30E-12	1.09E-10
rno-mir-148a-3p	-2.160846398	0.39053886	3.15E-08	3.19E-07
rno-mir-150-5p	-4.756016827	0.47496771	1.33E-23	9.79E-22
rno-mir-29a-3p	-1.066129721	0.35318518	0.00253934	0.01166509
rno-mir-23b-3p	-1.275657192	0.39567794	0.00126423	0.00640833
rno-mir-27a-3p	-1.389263014	0.30383009	4.82E-06	3.94E-05
rno-mir-192-5p	-8.482964793	0.72813939	2.29E-31	3.37E-29
rno-mir-10a-5p	-2.503339037	0.38551637	8.39E-11	1.17E-09
rno-mir-139-5p	-5.302658502	0.46385312	2.90E-30	2.84E-28
rno-mir-126a-3p	-8.239730125	0.52015203	1.62E-56	4.77E-54
rno-mir-99a-5p	-1.516216558	0.28863543	1.50E-07	1.37E-06
rno-mir-26a-5p	-2.092000928	0.2161814	3.77E-22	1.85E-20
rno-mir-122-5p	-7.382613566	0.77540318	1.72E-21	7.20E-20

Supplementary Table 5- miR-150 target genes and their function

Ortholog of target gene	Gene name
**MYB**	v-myb avian myeloblastosis viral oncogene homolog
**TADA1**	transcriptional adaptor 1
**ABCB9**	ATP-binding cassette, sub-family B (MDR/TAP), member 9
**FTO**	fat mass and obesity associated
**TMEM41A**	transmembrane protein 41A
**KLHL12**	kelch-like family member 12
**HILPDA**	hypoxia inducible lipid droplet-associated
**NKX2-4**	NK2 homeobox 4
**MBD6**	methyl-CpG binding domain protein 6
**GRIPAP1**	GRIP1 associated protein 1
**SP1**	Sp1 transcription factor
**NR2F2**	nuclear receptor subfamily 2, group F, member 2
**FOXD3**	forkhead box D3
**DYRK1A**	dual-specificity tyrosine-(Y)-phosphorylation regulated kinase 1A
**MTCH2**	mitochondrial carrier 2
**PAX5**	paired box 5
**IGF2BP1**	insulin-like growth factor 2 mRNA binding protein 1
**PIK3AP1**	phosphoinositide-3-kinase adaptor protein 1
**CDKN1B**	cyclin-dependent kinase inhibitor 1B (p27, Kip1)
**EGR2**	early growth response 2
**PTP4A1**	protein tyrosine phosphatase type IVA, member 1
**ADIPOR2**	adiponectin receptor 2
**AGO1**	argonaute RISC catalytic component 1
**DDB2**	damage-specific DNA binding protein 2, 48kDa
**PKP4**	plakophilin 4
**PSEN1**	presenilin 1
**ZEB1**	zinc finger E-box binding homeobox 1
**TLR5**	toll-like receptor 5
**H1F0**	H1 histone family, member 0
**UBR1**	ubiquitin protein ligase E3 component n-recognin 1
**AKT3**	v-akt murine thymoma viral oncogene homolog 3
**EIF4E**	eukaryotic translation initiation factor 4E
**UST**	uronyl-2-sulfotransferase
**SIM1**	single-minded homolog 1 (Drosophila)
**FOXP1**	forkhead box P1

**RSF1**	remodeling and spacing factor 1
**EP300**	E1A binding protein p300
**PPP2CB**	protein phosphatase 2, catalytic subunit, beta isozyme
**CALU**	calumenin
**HNRNPH3**	heterogeneous nuclear ribonucleoprotein H3 (2H9)
**GABRG2**	gamma-aminobutyric acid (GABA) A receptor, gamma 2
**CC2D1B**	coiled-coil and C2 domain containing 1B
**NCOA1**	nuclear receptor coactivator 1
**KIAA0087**	KIAA0087
**KIAA1549L**	KIAA1549-like
**ZBTB4**	zinc finger and BTB domain containing 4
**GTF3C2**	general transcription factor IIIC, polypeptide 2, beta 110kDa
**LETMD1**	LETM1 domain containing 1
**MMP14**	matrix metallopeptidase 14 (membrane-inserted)
**LMO4**	LIM domain only 4
**CELSR3**	cadherin, EGF LAG seven-pass G-type receptor 3
**SRCIN1**	SRC kinase signaling inhibitor 1
**UBFD1**	ubiquitin family domain containing 1
**ETF1**	eukaryotic translation termination factor 1
**ITSN1**	intersectin 1 (SH3 domain protein)
**NR1D2**	nuclear receptor subfamily 1, group D, member 2
**USP13**	ubiquitin specific peptidase 13 (isopeptidase T-3)
**ROCK1**	Rho-associated, coiled-coil containing protein kinase 1
**EPHB2**	EPH receptor B2
**APC**	adenomatous polyposis coli
**PAPD5**	PAP associated domain containing 5
**ACVR1C**	activin A receptor, type IC
**UPF1**	UPF1 regulator of nonsense transcripts homolog (yeast)
**GSTO2**	glutathione S-transferase omega 2
**TAF1**	TAF1 RNA polymerase II, TATA box binding protein (TBP)-associated factor, 250kDa
**CHD2**	chromodomain helicase DNA binding protein 2
**FOXO4**	forkhead box O4
**IL1RAPL1**	interleukin 1 receptor accessory protein-like 1
**PFN2**	profilin 2
**RC3H1**	ring finger and CCCH-type domains 1
**FGFR3**	fibroblast growth factor receptor 3
**EDA**	ectodysplasin A

**FZD4**	frizzled family receptor 4
**TET3**	tet methylcytosine dioxygenase 3
**CAPN6**	calpain 6
**CPEB4**	cytoplasmic polyadenylation element binding protein 4
**GLIS3**	GLIS family zinc finger 3
**PRKCA**	protein kinase C, alpha
**MAP3K9**	mitogen-activated protein kinase kinase kinase 9
**NFASC**	neurofascin
**COL4A4**	collagen, type IV, alpha 4
**MMP16**	matrix metallopeptidase 16 (membrane-inserted)
**STEAP3**	STEAP family member 3, metalloreductase
**PARVA**	parvin, alpha
**PAPPA**	pregnancy-associated plasma protein A, pappalysin 1
**CPD**	carboxypeptidase D
**CMIP**	c-Maf inducing protein
**DST**	dystonin
**FBXW11**	F-box and WD repeat domain containing 11
**UBE3B**	ubiquitin protein ligase E3B
**MAP3K12**	mitogen-activated protein kinase kinase kinase 12
**ZDHHC3**	zinc finger, DHHC-type containing 3
**GLG1**	golgi glycoprotein 1
**PA2G4**	proliferation-associated 2G4, 38kDa
**PLXNC1**	plexin C1
**RARG**	retinoic acid receptor, gamma
**GSK3B**	glycogen synthase kinase 3 beta
**GID4**	GID complex subunit 4
**MGA**	MGA, MAX dimerization protein
**CBX8**	chromobox homolog 8
**ATP8A2**	ATPase, aminophospholipid transporter, class I, type 8A, member 2
**PLXNB1**	plexin B1
**USP49**	ubiquitin specific peptidase 49
**BCL9L**	B-cell CLL/lymphoma 9-like
**HIF1A**	hypoxia inducible factor 1, alpha subunit (basic helix-loop-helix transcription factor)
**THBS2**	thrombospondin 2
**ABI2**	abl-interactor 2
**FNDC5**	fibronectin type III domain containing 5
**PLRG1**	pleiotropic regulator 1

**GHSR**	growth hormone secretagogue receptor
**IRF2BP2**	interferon regulatory factor 2 binding protein 2
**NOTCH3**	Notch Receptor 3; receptor for membrane-bound ligands Jagged1, Jagged2 and Delta1 to regulate cell-fate determination
**MUC4**	Major constituent of mucus, may play a role in tumour progression
**VEGFA**	Vascular Endothelial Growth Factor A, member of the PDGF/VEGF growth factor family
**P2RX7**	Purinergic Receptor P2X 7, a member of purinoceptors for ATP; functions as a ligand-gated ion channel
**IGF2**	Insulin-like growth factor 2
**ZEB1**	Zinc Finger E-Box Binding Homeobox 1, zinc finger transcription factor
**CXCR4**	C-X-C Motif Chemokine Receptor 4; chemokine receptor specific for stromal cell-derived factor-1

Supplementary Table 6 – ALD patient characteristics

Gender	Age (years)	Bilirubin (umol/L)	Platelets (x10^9 per litre)	ALT (U/L)	PT (secs)	Creatinine (umol/L)	Sodium (mmol/L)
M	48	49	79	18	16	68	140
M	63	39	215	21	17	74	132
M	56	168	130	67	12	73	138
M	62	20	72	22	14	92	139
M	60	11	179	25	11	76	144
M	64	25	155	35	11	208	131
M	55	70	65	23	23	49	143
M	47	26	133	33	14	83	135
M	61	11	245	51	11	83	138
M	58	23	103	17	13	145	129
F	65	7	167	50	13	71	141
M	59	36	115	24	14	49	139
M	63	25	123	14	13	107	142
M	75	3	221	13	11	124	140
F	64	4	588	19	12	47	140
M	58	14	259	28	15	55	136
F	69	12	174	30	12	77	142
M	47	22	113	39	12	62	141
M	34	15	127	70	11	109	145
M	68	11	116	17	14	66	141

Supplementary Table 7 – Control patient characteristics

Gender	Age (years)
M	45
M	57
F	31
M	33
F	28
F	34
M	29
F	28
M	34
M	30

Western blots- full images

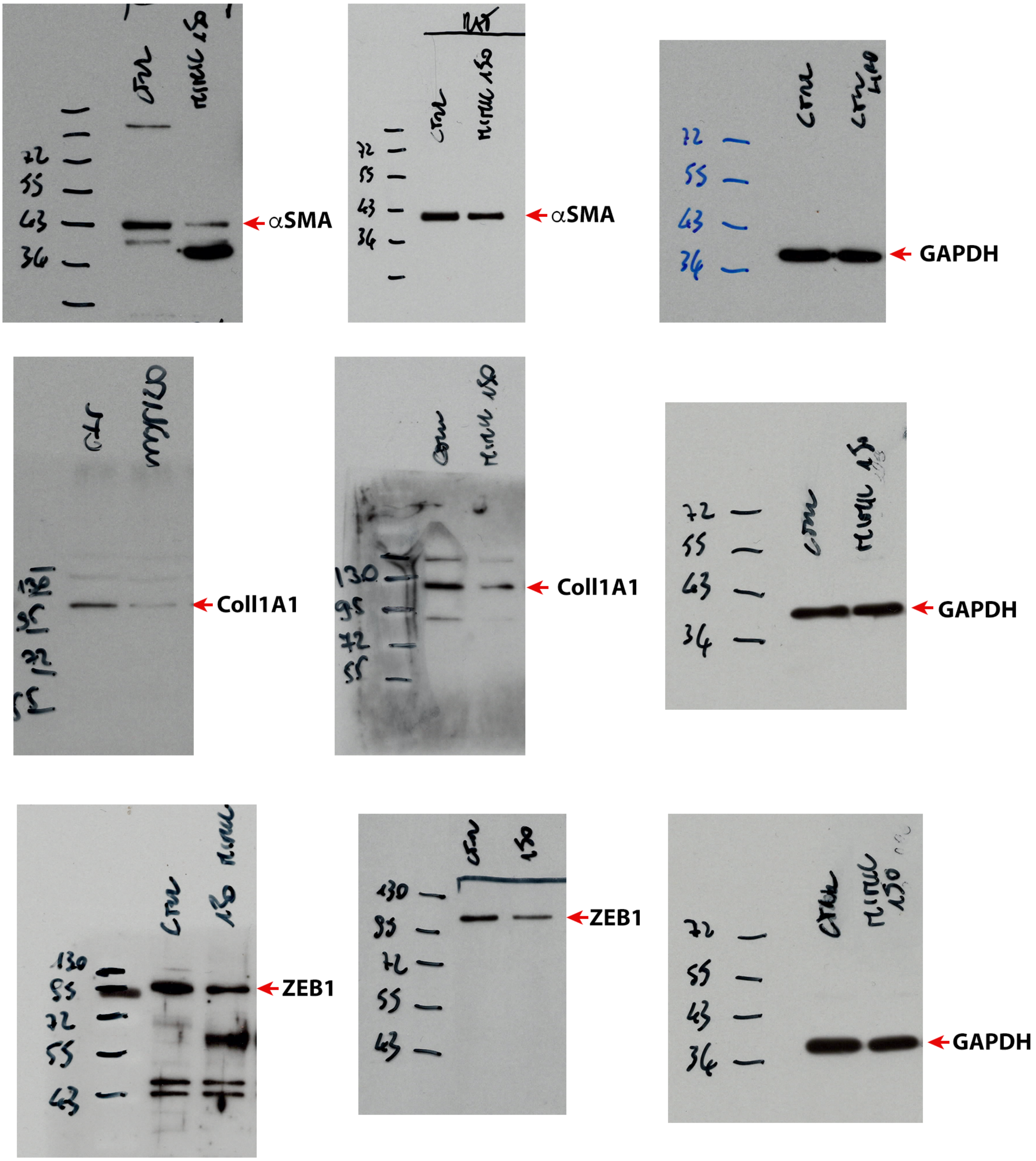

Supplement: Supplementary file 1 — Supplementary information. [file 41598_2020_78776_MOESM1_ESM.pdf]
